# Tracing the footprints of a moving hybrid zone under a demographic history of speciation with gene flow

**DOI:** 10.1111/eva.12795

**Published:** 2019-04-29

**Authors:** Mitra Menon, Erin Landguth, Alejandro Leal‐Saenz, Justin C. Bagley, Anna W. Schoettle, Christian Wehenkel, Lluvia Flores‐Renteria, Samuel A. Cushman, Kristen M. Waring, Andrew J. Eckert

**Affiliations:** ^1^ Integrative Life Sciences Virginia Commonwealth University Richmond Virginia; ^2^ School of Public and Community Health Sciences University of Montana Missoula Montana; ^3^ Programa Institucional de Doctorado en Ciencias Agropecuarias y Forestales Universidad Juárez del Estado de Durango Durango Mexico; ^4^ Department of Biology Virginia Commonwealth University Richmond Virginia; ^5^ Rocky Mountain Research Station USDA Forest Service Fort Collins Colorado; ^6^ Instituto de Silvicultura e Industria de la Madera Universidad Juarez del Estado de Durango Durango Mexico; ^7^ Department of Biology San Diego State University San Diego California; ^8^ Rocky Mountain Research Station USDA Forest Service Flagstaff Arizona; ^9^ School of Forestry Northern Arizona University Flagstaff Arizona

**Keywords:** CDMetaPOP, cline analysis, conifers, forest management, hybrid zone movement, hybrid zones, range dynamics

## Abstract

A lack of optimal gene combinations, as well as low levels of genetic diversity, is often associated with the formation of species range margins. Conservation efforts rely on predictive modelling using abiotic variables and assessments of genetic diversity to determine target species and populations for controlled breeding, germplasm conservation and assisted migration. Biotic factors such as interspecific competition and hybridization, however, are largely ignored, despite their prevalence across diverse taxa and their role as key evolutionary forces. Hybridization between species with well‐developed barriers to reproductive isolation often results in the production of offspring with lower fitness. Generation of novel allelic combinations through hybridization, however, can also generate positive fitness consequences. Despite this possibility, hybridization‐mediated introgression is often considered a threat to biodiversity as it can blur species boundaries. The contribution of hybridization towards increasing genetic diversity of populations at range margins has only recently gathered attention in conservation studies. We assessed the extent to which hybridization contributes towards range dynamics by tracking spatio‐temporal changes in the central location of a hybrid zone between two recently diverged species of pines: *Pinus strobiformis* and *P. flexilis*. By comparing geographic cline centre estimates for global admixture coefficient with morphological traits associated with reproductive output, we demonstrate a northward shift in the hybrid zone. Using a combination of spatially explicit, individual‐based simulations and linkage disequilibrium variance partitioning, we note a significant contribution of adaptive introgression towards this northward movement, despite the potential for differences in regional population size to aid hybrid zone movement. Overall, our study demonstrates that hybridization between recently diverged species can increase genetic diversity and generate novel allelic combinations. These novel combinations may allow range margin populations to track favourable climatic conditions or facilitate adaptive evolution to ongoing and future climate change.

## 1 INTRODUCTION

The rate and direction of change in species range margins is determined by standing levels of genetic diversity, biotic and abiotic factors, and an interaction between genotypes and the environment (Sexton, McIntyre, Angert, & Rice, 2009). The “centre–periphery hypothesis” (CPH) is a long‐standing ecological hypothesis that is used to explain how the above‐mentioned factors influence range margins of species (Brown, 1984). Specifically, the CPH states that populations at the core of a species range are often at carrying capacity, whereas populations near the range margins are also likely near the margins of their ecological niche and tend to exhibit lower genetic diversity thereby limiting further range expansion (Eckert, Samis, & Lougheed, 2008; Lawton, 1993). The CPH makes key assumptions about the fundamental niche being similar across the geographical range of a species, and that climate optima are stable over time (Pironon et al., 2017; Sheth & Angert, 2018). Efforts to predict changes in range margins via ecological niche modelling (ENM) are now incorporating within‐species variation in the fundamental niche and genetic structure (Ikeda et al., 2017; Malone, Schoettle, & Coop, 2018). Still, biotic factors such as competition, tolerance to insects and pathogens and hybridization with a closely related species are often neglected in predictive modelling (but see Engler, Rödder, Elle, Hochkirch, & Secondi, 2013; Pollock et al., 2014). In particular, hybridization‐induced introgression is often considered a threat to biodiversity, as on one hand it can cause dilution of the local gene pool (Fitzpatrick et al., 2010) and can generate offspring with reduced fitness levels. On the other hand, introgression can increase genetic diversity, specifically in range margin populations, thus creating deviations from the patterns of genetic diversity expected under the CPH. Moreover, introgression often facilitates colonization of novel habitats by bringing together novel allelic combinations not seen in the range of either parental species (Abbott, Barton, & Good, 2016; Rieseberg et al., 2007; Stebbins, 1959). Mounting evidence for introgression facilitating evolutionary and ecological diversification in several taxa, such as cichlid fishes (Meier et al., 2017), *Saccharomyces* yeast (Stelkens, Brockhurst, Hurst, & Greig, 2014), conifers (de Lafontaine, Prunier, Gérardi & Bousquet, 2015), Darwin's finches (Lamichhaney et al., 2018) and even hominids (Jagoda et al., 2017), indicates its importance as an evolutionary process.

For parapatrically distributed species as well as recently diverged species, genome‐wide introgression can cause range shifts due to its effect on standing levels of genetic diversity (Hamilton & Miller, 2016; Pfenning, Kelly, & Pierce, 2016). Such hybridization‐induced changes in range margins tend to leave signatures of spatio‐temporal shifts in the location of hybrid zones. Spatio‐temporal dynamics of hybrid zones can be driven by varied processes such as interspecific competition, changes in population size, dynamic features of the landscape and varying selection pressures (Barton & Hewitt, 1985; Buggs, 2007; Pfenning et al., 2016). Whether these processes will cause a hybrid zone to experience asymmetric expansion, bidirectional expansion or contraction will additionally depend on the divergence history and life history characteristics of the hybridizing species. For instance, introgression is often considered to be a factor causing range contraction of native species (Todesco et al., 2016). However, such hybridization between native and invasive species may not be of concern if populations of the native species are not highly fragmented at the region of contact and are ecologically differentiated from the niche space that is suitable for hybrids (Currat, Ruedi, Petit, & Excoffier, 2008). Further, the hybrid zone dynamics literature has focussed on divergence histories involving secondary contact while those with punctuated gene flow or continual gene flow during divergence are largely missing.

The long‐term consequences of hybridization are rarely explored due to the paucity of field records describing the locations and composition of hybrid populations across generations (Britch, Cain, & Howard, 2008; Buggs, 2007; Taylor, White, et al., 2014). This shortcoming is often overcome by utilizing the mathematical theory of clines to assess coincidence in cline centres between nuclear and mitochondrial genomic data (Krosby & Rohwer, 2009; Souissi, Bonhomme, Manchado, Sfar, & Gagnaire, 2018), as well as between genetic markers and morphological traits associated with reproductive isolation (Arntzen, de Vries, Canestrelli, & Martínez‐Solano, 2017; Gay, Crochet, Bell, & Lenormand, 2008; Martin & Cruzan, 1999; Rohwer, Bermingham, & Wood, 2001). As such, the lack of coincidence in cline centre across different datasets occurs due to nonequilibria between drift and selection and is often referred to as shifts in species range margins or in the central location of the hybrid zone. This signature of hybrid zone movement, as seen through noncoincident cline centres, can help forecast the rate and direction of change in species range margins (Walsh, Shriver, Olsen, & Kovach, 2016), thereby streamlining conservation efforts. Although hybrid zone movement is ubiquitous across systems, the eco‐evolutionary processes at play are not well understood. Here, we overcome this hurdle by combining genomic and geographic cline analyses with individual‐based spatial simulations of a hybrid zone. We utilize two species of hybridizing white pines inhabiting mountainous regions in western North America to assess hybrid zone movement under a history of continuous gene flow and ecological divergence (Menon et al., 2018b). Besides being key components of the montane ecosystems in western North America (Looney & Waring, 2013; Windmuller‐Campione & Long, 2016), most sister species of pines exhibit weak isolating barriers (Critchfield, 1986), rendering them useful to study the interaction between adaptive and neutral introgression in driving hybrid zone dynamics.

**Figure 1:**
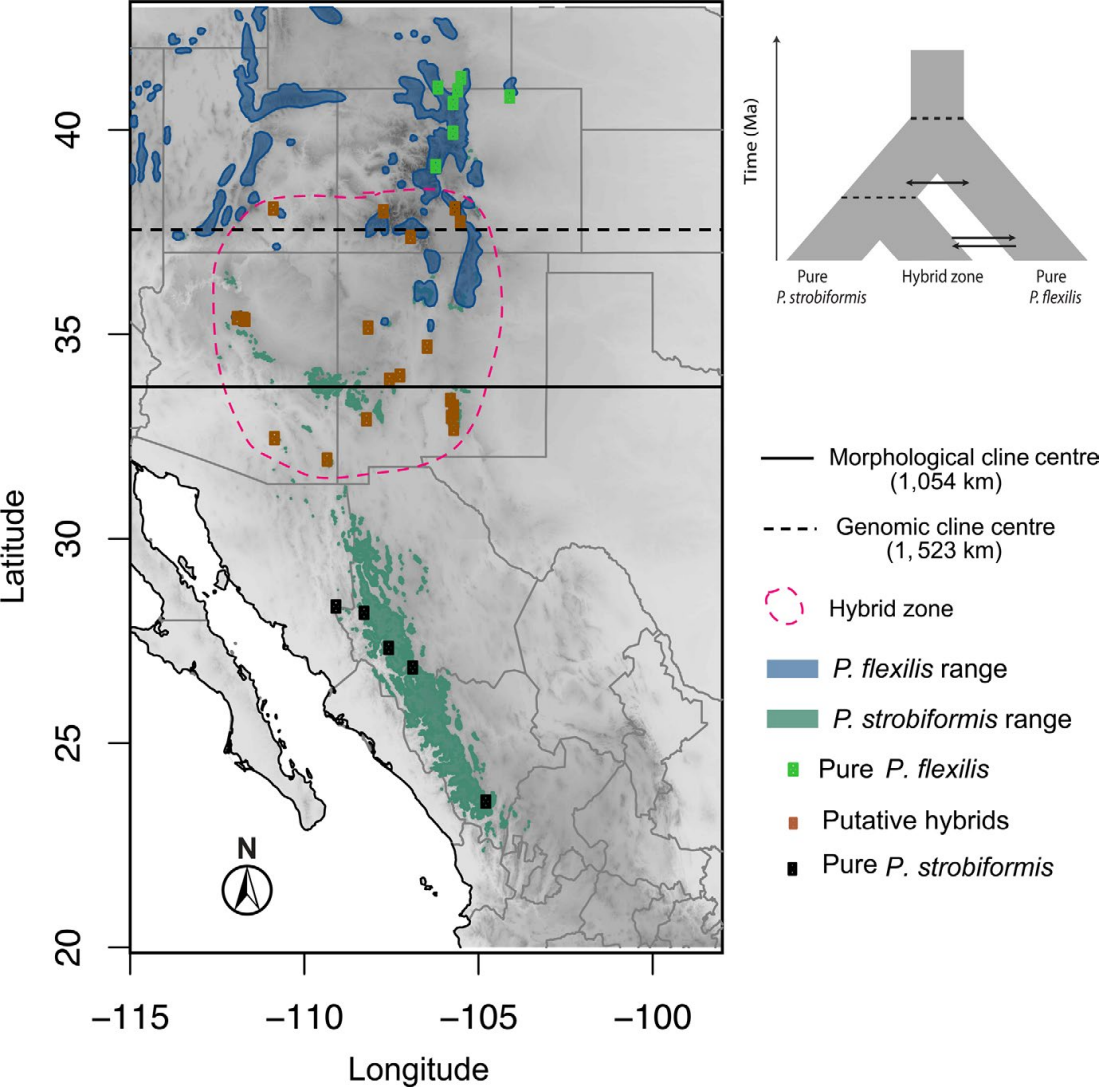
Map of sampled populations (squares) with overlaid polygons representing the geographical range of *Pinus strobiformis* (green) (obtained from Shirk et al., 2018) and *P. flexilis* (blue). The two horizontal lines represent the geographic locations of the cline centre as estimated from morphological (continuous line) and genomic data (dashed line). The dashed oval represents the full extent of the hybrid zone (as defined here). The inset figure shows the best fit demographic model from Menon et al. (2018b), indicating a history of divergence with gene flow and contemporary gene flow only between *P. flexilis* and the hybrid zone

*Pinus flexilis* E. James is distributed from northern Arizona and New Mexico in the southwestern United States to central Alberta, Canada, while *P. strobiformis* Engelm. ranges from southern Arizona and New Mexico to Jalisco in southern Mexico. The hybrid zone between *P. strobiformi*s and *P. flexilis* was first described by Engelmann in 1971 as extending across northern Arizona and north‐central New Mexico. This has recently been corroborated by morphological and genetic data (Bisbee, 2014; Menon et al., 2018b; Tomback, Samano, Pruett, & Schoettle, 2011). Both species grow at moderate to high elevations, but are divergent when characterized within multivariate niche space (Menon et al., 2018b; Moreno‐Letelier, Ortíz‐Medrano, & Piñero, 2013). This multivariate niche space is represented by a combination of drought intensity and duration or magnitude of sub‐zero temperatures, such that *P. strobiformis* occurs in areas of relatively higher drought and fewer days of sub‐zero temperatures when compared to *P. flexilis*. Despite the ecological niche divergence and a long history of divergence with gene flow, the two species lack strong isolating barriers and the hybrid zone continues to exchange genes only with *P. flexilis* (Figure 1; Menon et al., 2018b). The absence of strong isolating barriers is supported by field observations indicating the presence of populations containing trees with mixed ancestry being proximal to trees with pure parental characteristics (Steinhoff & Andresen, 1971) and by a genome‐wide dataset demonstrating the lack of loci associated with reproductive isolation (Menon et al., 2018b). These observations lead to two possible implications for the hybrid zone dynamics in this system. First, given a history of divergence with gene flow, the *P. strobiformis–P. flexilis* hybrid zone could be relatively spatially stable in comparison with recent hybrid zones or hybrid zones formed by secondary contact (Barton & Hewitt, 1985). Second, the ongoing introgression from *P. flexilis* into the hybrid or *P. strobiformis* genomic background could cause spatio‐temporal movement of the hybrid zone. We hypothesized that the latter is more likely the case given the spatial distribution of the hybrid zone on a fragmented landscape facilitating a higher rate of neutral introgression from *P. flexilis* than vice versa and the possible adaptive introgression of freeze‐related loci that may facilitate northward hybrid zone movement due to preference for cooler climatic conditions in this group of pines (Frankis, 2009; Larson & Moir, 1987; Moreno‐Letelier et al., 2013; Shirk et al., 2018). If our hypothesis holds true, then, management of hybrid populations should be prioritized, as these regions may contain novel allelic combinations that could make trees resilient by facilitating northward range expansion or by providing the raw material to adapt to rapidly changing climatic conditions.

To test our hypothesis, we combined information from empirical analyses of genetic and morphological data with spatial simulations of hybrid zones. Specifically, we address three questions that help us test distinguish the hypothesis: (1) Can we detect a signature of movement in the *P. strobiformis*–*P. flexilis* hybrid zone that was formed under a history of divergence with gene flow? (2) How does the empirical data pattern compare to hybrid zone dynamics noted under other models of divergence common in the literature? and (3) If hybrid zone movement is noted in the empirical dataset, can the magnitude of movement be explained purely by demographic processes? We address question 1 and question 2 by comparing geographic cline centre estimates between the two empirical datasets (genomic and morphological) and then assessing changes in geographic cline centre across different simulated scenarios. To address question 3, we combine results from genomic and geographic cline analyses within a contingency table along with linkage disequilibrium (LD) variance partitioning. Since our simulated dataset does not assume any form of selection, comparing temporal change in LD and relative shifts in the geographic cline centre between the empirical and simulated dataset proved useful to address question 2. Our results shed light on the role of introgression in facilitating hybrid zone movement, beyond what is likely to occur due to differences in regional population sizes. Thus, we emphasize the utility of hybrid zone populations as a key conservation resource owing to the value they provide for assisted migration or adaptation to rapidly changing climatic conditions.

## 2 METHODS

### 2.1 Data generation

We subsampled 34 of the 55 populations from Menon et al. (2018b) in order to match the locations for which morphological data were available (details under morphological dataset, Figure 1 and Figure S1). This resulted in a dataset of 332 trees with 3–10 individuals per population. For *P. strobiformis*, we sampled 21 populations, of which 16 were from the putative hybrid zone and 5 from pure parental populations. In Menon et al. (2018b), the pure populations of *P. strobiformis* were referred to as the “Core” while the hybrid zone populations were referred to as the “Periphery.” For *P. flexilis*, we sampled 13 populations, of which 6 were closer to the hybrid zone and 7 were from pure parental populations outside the hybrid zone.

#### 2.1.1 Morphological data set

*Pinus strobiformis* morphological data were obtained from 40 and 39 natural populations (3–8 trees/population) in Mexico and the United States, respectively (Figure S1). Each population was separated by a minimum distance of 50 km, and each tree within a stand was separated by a minimum distance of 50–70 m (Goodrich, Waring, & Flores‐Renteria, 2018). Mean cone length (cm) and mean seed weight for 10 filled seeds (g) were obtained from 10 air‐dried ripe cones per tree with no visible signs of insect or disease damage. Further information about data collection and processing of the samples are detailed in Leal‐Saenz et al., 2018). For *P. flexilis*, morphological data were obtained from 13 natural populations (5–10 trees/population) in Colorado and southern Wyoming (Figure 1). Choice of populations and trees along with the protocol used for cone and seed measurements were similar to those for *P. strobiformis*.

The use of cone length and seed weight to assess hybrid zone movement is based on their association with fitness and specifically with reproductive output in conifers (Mosseler et al., 2000). Further, they are often used as diagnostic traits to distinguish pine species (Bisbee, 2014; Frankis, 2009; Leal‐Saenz et al., 2018). Of the 55 populations in the genetic dataset, only 24 had exactly matching coordinates with the morphological dataset. To increase our sample size, we averaged the morphological data from populations that were within a 5‐km radius of each missing population in our genetic dataset. Through this approach, we were able to add 10 populations, resulting in a total of 34 populations in the final dataset (Figure 1). The choice of 5 km is based on pollen dispersal kernels and paternity analysis in pines demonstrating pollen viability even at 41 km away from its source of origin (Williams, 2010). Hence, individual trees within a 5‐km radius can be considered closely related to each other. To bolster this argument, we utilized an independent genetic dataset and estimated individual relatedness for trees along gradients of geographical distance from 0 to 800 km within the hybrid zone by using the probability of sharing two alleles implemented within RelateAdmix (Moltke & Albrechtsen, 2014). Our assessment indicated that relatedness did not change much across this range of geographical distances (results not shown), thereby justifying the use of a 5‐km threshold to group populations.

#### 2.1.2 Genetic dataset

Genotypic data were taken from Menon et al. (2018b). In brief, ddRADseq libraries following Parchman et al. (2012) were generated from total genomic DNA extracted from needle tissue of 445 individuals across 55 populations. These were downsampled to 332 individuals across 34 populations, following the approach detailed above. Each library contained up to 96 multiplexed samples that were individually digested using *EcoR1* and *Mse1* restriction enzymes. Fragments in the 300–400 bp size range were selected post‐PCR and sequenced on Illumina HiSeq 2500 at the Nucleic Acids Research Facility located in Virginia Commonwealth University. An initial set of single nucleotide polymorphisms (SNPs) were obtained by processing the output FASTQ file using the dDocent bioinformatics pipeline (Puritz, Hollenbeck, & Gold, 2014). These SNPs were subsequently filtered using custom Python scripts to yield a final set of 51,633 SNPs.

#### 2.1.3 Simulation data set

We used CDMetaPOP v1.14 (Landguth, Bearlin, Day, & Dunham, 2016) to simulate the spatial movement of individuals under varying modes of speciation. Briefly, CDMetaPOP is a spatially explicit and individual‐based eco‐genetic model of meta‐population dynamics that simulates demographic and genetic processes as interactions between individuals located across a number of patches containing meta‐populations (hereafter, “groups”). Our landscape included three groups: *P. strobiformis*, hybrid zone and *P. flexilis*. We matched the spatial set‐up (location and extent of each group), dispersal parameters and the degree of genetic divergence among groups within CDMetaPOP to empirical estimates appropriate to our study system. Further details about parameterization and landscape set‐up are listed in Appendix S1 and Table S1.

**Figure 2:**
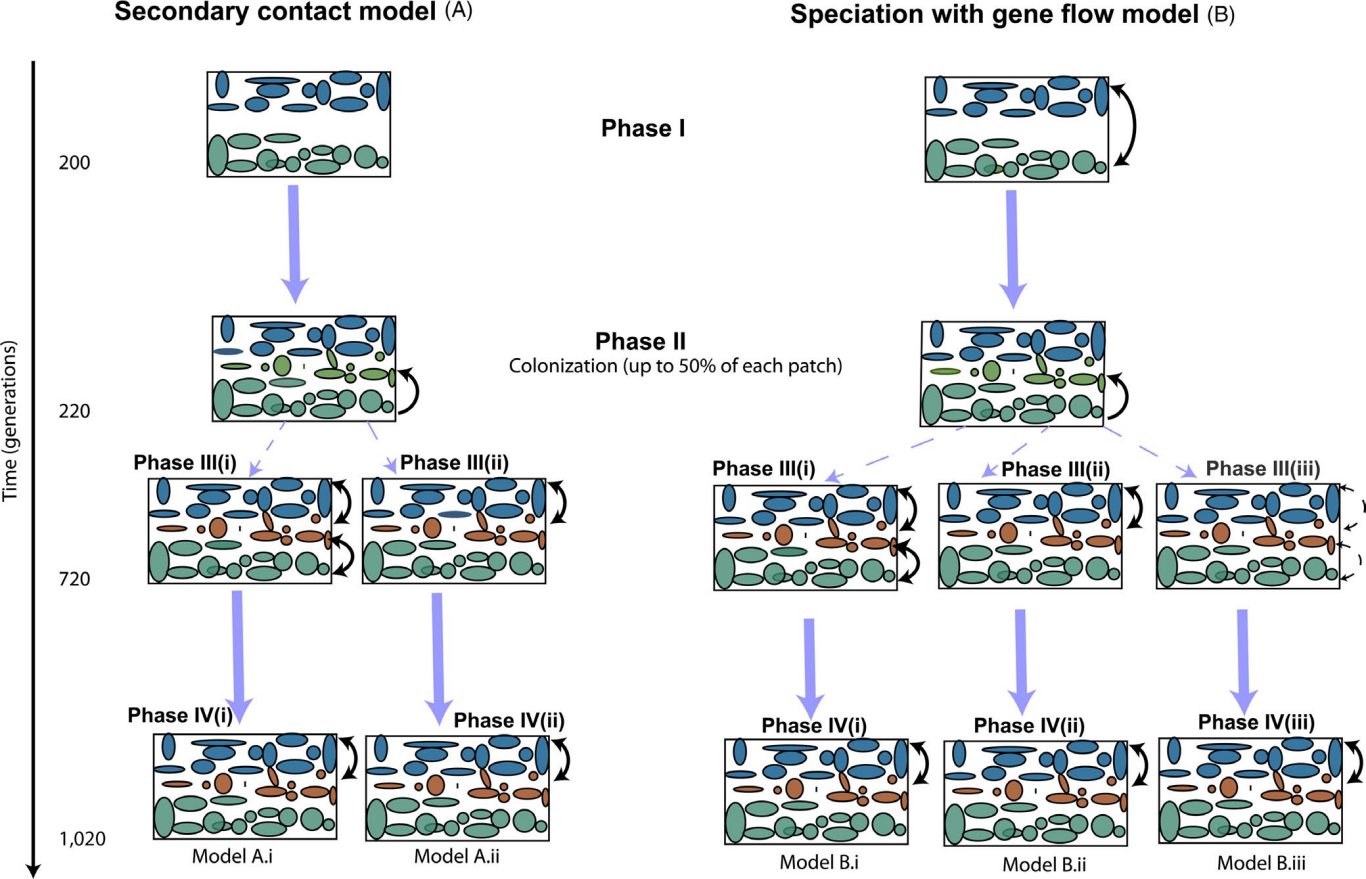
Layout for the simulation framework implemented in CDMetaPOP. The three colours (blue, brown and green) correspond to patches representing *P. flexilis*, hybrid zone and *P. strobiformis*. Arrows represent the directionality of dispersal between groups, with the dashed arrows indicating dispersal reduced by 50%. The scenario names are listed below Phase IV. Patch and movement parameters are detailed in Table S1

In order to match the simulation framework to the empirical data set, we divided the simulation workflow into three phases across the two models of speciation shown in Figure 2. Phase I only included two groups, pure *P. strobiformis* (green patches) and pure *P. flexilis* (blue patches) and represents the initial process of divergence between them. Patches in the middle were available but not yet occupied. We allowed the cycle of birth, migration, reproduction and death to occur every year and continue for 200 nonoverlapping generations. Phase II was the colonization phase, during which individuals from pure *P. strobiformis* started colonizing empty patches in the middle of the landscape for 20 nonoverlapping generations (light green). Once each patch in the middle had attained 50% carrying capacity, the among‐group gene flow parameter was modified to generate hybrids and to incorporate the influence of spatially restricted gene flow in scenarios A.ii, B.ii and B.iii (Phase III: Figure 2 and Table S1). Phase III consisted of two scenarios that are common across the models of secondary contact (A) as well as models of speciation with gene flow (B). For scenario 1 (Phase III.i), the hybrid zone experienced bidirectional dispersal from both *P. flexilis* and *P. strobiformis*, whereas for scenario 2 (Phase III.ii) the hybrid zone experienced bidirectional dispersal only from *P. flexilis*, in accordance with the best fit demographic model in Menon et al. (2018b). For the speciation with gene flow model, we added a third scenario (PhaseIII.iii) that was similar to Phase III.i but here among‐group dispersal was reduced by 50%. All scenarios in Phase III were run for 500 generations. Phase IV of our simulation was the same across all conditions, and only included bidirectional dispersal between the hybrid zone and *P. flexilis* for 300 generations. Parametrization for Phase IV was set in accordance with the contemporary pattern of gene flow as estimated from the best fit demographic model identified in Menon et al. (2018b). Overall, we had five scenarios at the end of Phase IV, of which scenarios A.i and A.ii were nested within the secondary contact model (Model A) and scenarios B.i, B.ii and B.iii were nested within the speciation with gene flow model (Model B). For each scenario, we performed 12 replicate runs.

To ensure that each of the scenarios mimicked the overall pattern of divergence in our empirical dataset, we estimated the overall levels of genetic differentiation (*F*_ST_) and differentiation among groups (*F*_CT_) using the hierarchical model implemented in HIERFSTAT (Goudet, 2005). Measures of genetic differentiation were obtained every 50 generation throughout our simulation to ensure that our divergence level matched the observed level of divergence. Our overall analyses indicated that the average value of *F*_ST _and *F*_CT _converged to the empirical estimates, specifically for scenarios that included some form of gene flow during Phase III (Table 1). Further details about the change in *F*_ST _and *F*_CT _values across time and across scenarios are presented in Appendix S2.

**Table 1:** Among group (mean *F*_CT_ ± 1*SE*) and among population within group (mean *F*_ST_ ± 1*SE*) genetic differentiation measures from Phase I to Phase III across Model A and Model B in the simulations

Model	Phase	*F*_ST_	*F*_CT_
(A) Secondary contact	I	0.024 ± 0.0001	0.014 ± 0.0002
	III(i)	0.009 ± 5.69e‐05	0.0004 ± 3.32e‐05
	III(ii)	0.026 ± 0.0006	0.018 ± 0.0001
	IV(i)	0.018 ± 0.0003	0.008 ± 0.0005
	IV(ii)	0.041 ± 0.0004	0.040 ± 0.0007
(B) Speciation with gene flow	I	0.013 ± 0.0001	0.002 ± 0.0001
	III(i)	0.009 ± 1.90e‐05	0.0004 ± 4.29e‐06
	III(ii)	0.021 ± 0.0004	0.013 ± 0.0006
	III(iii)	0.030 ± 0.0004	0.019 ± 0.0003
	IV(i)	0.019 ± 0.0002	0.008 ± 0.0003
	IV(ii)	0.034 ± 0.0003	0.032 ± 0.0004
	IV(iii)	0.037 ± 0.0003	0.031 ± 0.0004

### 2.2 Data analysis

#### 2.2.1 Geographic and genomic cline analyses

The geographic cline analysis was conducted for both empirical (genomic and morphological dataset) and the simulated datasets; however, genomic cline analysis was conducted only for the empirical dataset. We first compared the geographic cline centre estimates between the genomic and the morphological dataset to determine whether the empirical data supported hybrid zone movement (hypothesis 2). Next, we used simulations to assess the temporal change in geographic cline centre across various demographic scenarios with two common types of divergence histories. Finally, we combined the results from genomic and geographic cline analyses to assess whether hybrid zone movement can be expected in the absence of selection.

We estimated great circle geographical distances (km) from the southernmost population to all other sampled populations using the package Geosphere v‐1.5.7 (Hijmans, 2017) in R‐v.3.3.0 (R Core Team, 2017). Prior to fitting clines to the morphological dataset, we conducted a Shapiro–Wilk's test for normality in R and visualized the *Q*–*Q* plot of cone length and seed weight across the pure parental populations of both species. Both seed weight and cone length were normally distributed (*P. strobiformis* seed weight *p*‐value = 0.44, and cone length *p*‐value = 0.07; *P. flexilis* seed weight *p*‐value = 0.38, and cone length *p*‐value = 0.81) and hence satisfied assumptions of cline models (Barton & Gale, 1993). For the genetic data, we conducted two sets of geographical cline analyses—one for the global admixture coefficients estimated using fastSTRUCTURE (Raj, Stephens, & Pritchard, 2014) based *Q*‐scores from all 51,633 SNPs, and a second set using the allele frequency of each of the nearly diagnostic SNPs (as in Wielstra et al., 2017). Nearly diagnostic SNPs were defined as those in the top 10% percentile of allele frequency difference between the pure parental ranges of both species (*n* = 4,857 SNPs).

We utilized the Metropolis–Hastings Markov chain Monte Carlo (MCMC) algorithm implemented within HZAR v:0.2.5 in R (Derryberry, Derryberry, Maley, & Brumfield, 2013) to conduct cline fitting. We ran six replicate cline models for each dataset with different random seeds, each having a chain length of 100,000 steps after a burn‐in of 10,000 steps. For each fitted model, the trace plot of each parameter estimate across replicate MCMC runs was examined to assess whether runs had converged to the same value. For the morphological data, we fit five models with varying exponential tail estimations (none, left‐only, right‐only, mirror tails and both tails estimated separately) and assessed their fit using the corrected Akaike information criteria (AICc) model selection framework. For the best fit model, we obtained maximum‐likelihood estimates (MLEs) of geographical cline centre and cline width, as well as the 95% confidence interval (CI) given by ±2 log‐likelihood units (2 LLU) around the MLEs. For the *Q*‐score and individual allele frequency estimates from 4,857 nearly diagnostic SNP set, we fit 15 different cline models with varying combinations of the tail (5 possibilities detailed above) and scaling parameters (fixed at 0 & 1, estimated or observed values). The 15 different genetic cline models were compared against each other and also against a null model of no cline using AICc model selection. Geographic centre estimates from the best fit model were obtained for a total of 4,858 different cline models (1 global *Q*‐score + 4,857 nearly diagnostic SNPs). If the geographic cline centre estimates for the *Q‐score* and morphological dataset were noncoincident, it indicated a shift in the location of the hybrid zone towards the estimate obtained using the *Q‐score*.

For the simulated datasets, *Q*‐score estimates from fastSTRUCTURE at *K* = 2 were used to conduct the geographic cline analysis using a similar approach as detailed above. However here, the comparison was made across time rather than between *Q*‐score and the morphological dataset. We estimated the geographic cline centre across replicate runs for all scenarios starting at generation 500 for every 5 generations up to generation 520 and then every 50 generations up to generation 1,020. The geographic cline centres from the simulated scenarios were compared to the *Q*‐score estimate from the empirical dataset by assessing the percentage change in the cline centre relative to the total vertical spatial extent of the respective landscapes (simulated vs. empirical). We then examined whether the relative change noted was equal to or greater than the relative change noted in the empirical dataset. On one hand, if the relative change in the simulated scenarios matched the estimate for the empirical *Q*‐score, then genetic drift and gene flow alone could have facilitated the hybrid zone movement. On the other, if relative change was higher in our empirical dataset, then selection could be a key factor in driving hybrid zone movement.

To determine whether the lack of coincidence noted in the geographic cline analyses was a result of adaptive introgression or neutral patterns of introgression, we utilized the results from genomic cline analysis detailed in Menon et al. (2018b). This analysis was conducted only for the empirical dataset for each of the 4,857 nearly diagnostic SNPs, as a function of the given level of genomic admixture estimated within Bayesian genomic cline analyses (Gompert & Buerkle, 2012; Gompert, Parchman, & Buerkle, 2012). Estimates of genomic cline centre (*α*) were used to identify loci exhibiting exceptional introgression from *P. strobiformis* (negative *α*) or from *P. flexilis* (positive *α*) and to test whether the directionality of introgression was strongly associated with the *Q*‐score geographic cline centre or the morphological cline centre estimate. Asymmetric introgression is often reflective of hybrid zone movement and could aid an understanding of the relative roles of demographic processes and selection in driving this movement. Specifically, higher than expected association between the genomic and *Q*‐score geographic cline centre will indicate the role of adaptive introgression towards hybrid zone movement (question 3).

To determine the relative influence of genetic drift and selection on hybrid zone dynamics, we defined categories of genomic and geographic cline centre to conduct a 3 × 3 contingency test in R at a significance threshold of *α* = 0.01. For the geographic cline analyses, we classified the genomic cline centre for each of the 4,857 nearly diagnostic SNPs as coincident with either the *Q*‐score or morphological geographic cline centre, depending on whether it was within the confidence interval (i.e., 2 LLU) of the cline centre for either the *Q*‐score or morphological data. A category of “neither” was assigned to SNPs overlapping neither the *Q*‐score nor the morphological cline centres. For the *α* values, we classified the SNPs as positive outliers, negative outliers or not an outlier using the results from Menon et al. (2018b). Given ongoing gene flow only between *P. flexilis* and the hybrid zone (Figure 1 inset), we expected a strong association between the *Q*‐score cline centre and positive *α* outliers (positive genomic cline centre)*,* indicating that adaptive introgression from *P. flexilis* is promoting hybrid zone movement.

#### 2.2.2 Linkage disequilibrium

Elevated linkage disequilibrium (LD) can result from a variety of evolutionary processes. For instance, localities experiencing recent hybridization and/or ongoing range expansions are expected to have on average higher LD. This can be explained by large haplotype blocks created through recent hybridization or by bottlenecks following range expansions. Alternatively, selection on multiple loci involved in the maintenance of species boundaries could also elevate LD. Selection, however, would only increase the variance in LD rather than elevating the average value across all genotyped loci. Narrow hybrid zones often tend to have globally elevated LD due to the interaction between selection against hybrids with reduced fitness and constant parental dispersal (Barton & Hewitt, 1985; Mallet et al., 1990). For wider and older hybrid zones, as in this study, the pattern of LD could vary spatially and will depend on current patterns of hybridization and the strength of isolating barriers. For instance, Wang et al. (2011) and van Riemsdijk, Butlin, Wielstra, and Arntzen (2018) demonstrated the spatial change in LD as indicative of hybrid zone movement, such that an increase in LD is noted near the expanding front of the hybrid zone. However, in the absence of a linkage map, as is the case for several non‐model systems, partitioning of LD into among‐ and within‐population components (*D*_ST _& *D*_IS_; sensu Ohta, 1982) will be needed to document the relative importance of drift and selection in driving the hybrid zone movement. Specifically, if hybrid zone movement is noted in our other analyses, the LD variance partitioning will further help understand the relative importance of drift and selection in driving this movement. For instance, if neutral introgression is driving the noted movement, then we expect an increase in the ratio of *D*_IS_:*D*_ST_ towards the direction of hybrid zone movement. Prevalence of adaptive introgression could also increase *D*_ST_ *;* however, if the introgressed loci are globally favoured across all hybrid populations, it is unlikely to elevate *D*_ST_ while only *D*_IS_ will exhibit an increase at the expanding front.

To partition LD among and within groups arrayed along the latitudinal gradient of hybridization, we divided the 34 populations into 11 overlapping sets of four populations each, with an overlap of one population (Table S2). For each of the 11 sets obtained from the empirical data, we used the nearly diagnostic 4,857 SNPs, defined above under geographic cline analysis and estimated among‐ and within‐population LD (*D*_ST _& *D*_IS_) using Ohta's *D* statistics implemented in the R package, OhtaDstat (Beissinger et al., 2015). Similarly, for the simulated dataset, we utilized the nearly diagnostic SNPs (100 SNPs) at generation 1,020 (end of Phase IV) and at generation 300 (during Phase III) to examine changes in *D*_ST _and *D*_IS _across the simulated landscape and across time to assess the influence of hybrid zone movement on LD. The two time points were assessed to determine whether the spatial change in LD, if noted, was a result of landscape fragmentation alone or the combined result of landscape fragmentation and hybrid zone movement. The higher degree of fragmentation and lower population size within the hybrid zone could elevate drift and drive an increase in *D*_IS _. By holding the landscape configuration constant between generation 300 and generation 1,020 but altering only the pattern of introgression (Figure 2), we were able to disentangle the pure effect of landscape fragmentation from hybrid zone movement driven by neutral introgression.

## 3 RESULTS

### 3.1 Geographic and genomic cline analyses

**Figure 3:**
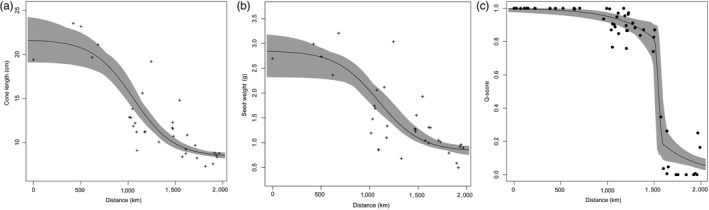
Empirical data‐based geographic cline as a function of distance from the southernmost population plotted for (a) cone length (b) seed weight and (c) *Q*‐score obtained from fastSTRUCTURE

Trees within the putative *P. strobiformis–P. flexilis* hybrid zone exhibited intermediate phenotypes with respect to cone length and seed weight. The best fit model for both cone length and seed weight did not include the exponential tail. The MLE of geographic cline centre and the 95% CI around it for seed weight and cone length were 1,100 km (980–1264 km) and 1,054 km (968–1132 km), respectively (Figure 3a,b; Table 2). The MLE of cline width for seed weight was 53 km wider than that of cone length (Table 2).

**Table 2:** Maximum‐likelihood estimates (MLEs) for geographic cline parameters and the 2 log‐likelihood unit (2 LLU) variation around each parameter

Data set	Centre (km)	2LLU (km)	Width (km)	2LLU (km)	AICc
Cone length	1,054	968–1,132	845	662–1,092	752.71
Seed weight	1,100	980–1,264	898	714–1,117	254.12
*Q*‐score	1,523	1,485–1,554	117	112–233	25.98

Using the *Q*‐score obtained from all 51,633 SNPs in fastSTRUCTURE, the MLE of geographic cline centre and width was 1,523 and 106 km, respectively (Figure 3c; Table 2). This best fit model included an exponential mirror tail fit and fixed scaling parameter set to 0 and 1 at either end of the cline. The 95% CI around the MLE of *Q*‐score cline centre did not overlap with the cline centre estimate for either morphological trait (Table 2). Overall, the average geographic cline centre estimate for *Q*‐score was shifted northward by 446 km relative to that of the morphological data, corresponding to a shift of 21% relative to the full vertical extent of the landscape. Of the 4,857 nearly diagnostic SNPs (defined in the Section 2), 1,266 were excluded from further analysis either due to the null model (no cline) being the best fit to the data (*n* = 977) or because the cline centre estimate was greater than the actual length of the transect used in our study (*n* = 289). For the remaining 3,590 SNPs, the mean (median) estimate of geographic cline centre was 1,358 km (1,484 km) ± 6 km (standard error, *SE*) units around the mean.

Of these 3,590 SNPs, the geographic cline centres for 608 were coincident with the *Q*‐score cline centre, whereas 523 had a cline centre coincident with the morphological trait centre. Of the 608 that were coincident with the *Q*‐score cline centre, 109 were positive *α* outliers (*P. flexilis* ancestry) and 325 were negative *α* outliers (*P. strobiformis* ancestry). Of the 523 SNPs that were coincident with the morphological trait centre, 56 were positive *α* outliers and 212 were negative *α* outliers. The 3 × 3 chi‐square contingency test for the association between *α* outlier categories and geographic cline centre categories was significant (*Χ* ^2^ = 76.727, *df* = 4, *p*‐value = 8.8 × 10^−16^). This significance was predominantly driven by loci that were not *α* outliers and exhibited a strong association with the morphological cline centre. Our results also indicated that SNPs overlapping with the *Q*‐score cline centre (i.e., exhibiting a northward shift) were strongly associated with positive *α* outliers (i.e., retention of *P. flexilis* ancestry). However, SNPs with geographic cline centres overlapping with the morphological data were likely to be identified as either negative *α* outliers (i.e., retention of *P. strobiformis* ancestry) or not an outlier with respect to *α* (Table 3).

**Table 3:** Percentage contribution of each category to the 3 × 3 contingency test using genomic cline centre (*α*) and geographic cline centre estimates. Values listed are the percentage contribution to the chi‐square statistic

	Positive *α* outlier (*P. flexilis* ancestry)	Negative *α* outlier (*P. strobiformis* ancestry)	Not outlier
Overlap *Q*‐score centre	**17.56**	0.18	*9.81*
Overlap morphological centre	*2.57*	*20.66*	**42.46**
Overlap neither	*1.76*	**3.61**	*2.33*

Note

Bold values indicate positive deviations from the expected value, and italicized values indicate negative deviations.

For both Models A and B in the spatial simulations, the geographic cline centre estimate stabilized across time and across replicates as we moved from high levels of dispersal to spatially restricted patterns of dispersal (Figure 5). Across these models, Phase III exhibited greater variation in the geographic cline centre estimate across generations relative to that exhibited during Phase IV. For the scenarios with spatially restricted dispersal (scenarios A.ii, B.ii and B.iii), Model A and Model B performed similarly and exhibited minimal variation in the geographic cline centre estimate across generations and replicates. At any given point in time, larger shifts in the cline centre were also accompanied by a greater degree of variation around the mean estimate (Figure 5). Only at the juncture of Phase III and Phase IV, across scenarios A.i and B.i, we noticed a mean relative shift in the geographic cline centre at or near the 21% northward shift noted in the empirical dataset. Moreover, for scenarios in the simulated dataset that matched the contemporary pattern of gene flow in our empirical dataset (i.e., A.ii, B.ii), we did not notice any time point that exhibited a geographic cline centre shift as sharp as or even near the 21% noted in the empirical dataset.

### 3.2 Linkage disequilibrium

**Figure 4:**
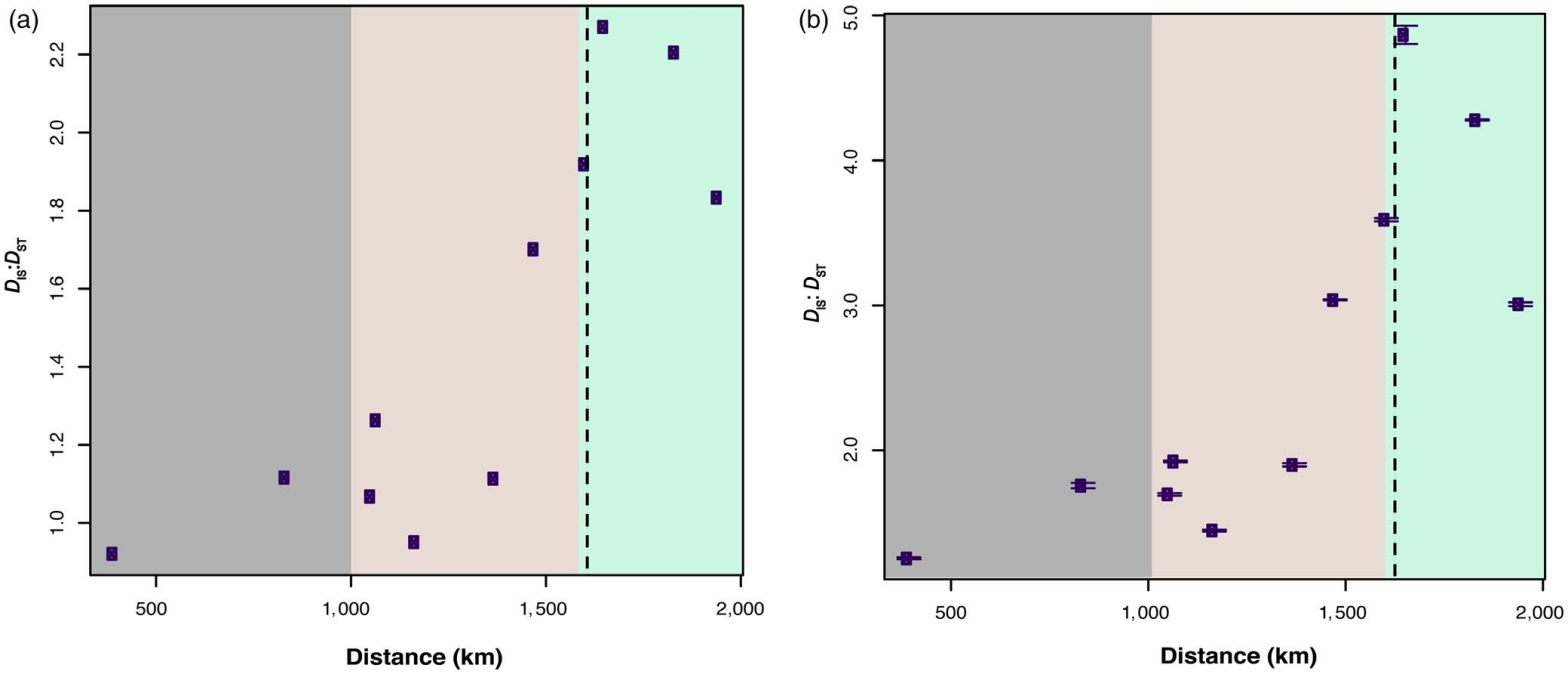
Change in the ratio of within‐population‐to‐among‐population variance components (*D*_IS_:*D*_ST_) for the empirical dataset plotted as a function of geographical distance for 11 sets of 4 populations (a) using 4,857 nearly diagnostic SNPs and (b) using 20 bootstrapped sets of nondiagnostic SNPs. The dotted vertical line represents the *Q*‐score geographic cline centre. The three colours represent the geographical location of pure *P. strobiformis* (grey), hybrid zone (brown) and pure *P. flexilis* (green)

For the 4,857 nearly diagnostic SNPs obtained from the empirical dataset, the ratio of within‐population‐to‐among‐population LD (*D*_IS_:*D*_ST_) exhibited a sigmoidal association with geographic distance from the southernmost population in our study (Figure 4a). Towards the southern edge, *D*_IS _was lower than *D*_ST_. As we move towards the northern margin of the hybrid zone, the ratio exhibits a steep positive increase, which is attributable to a sharp increase in *D*_IS _estimate while *D*_ST_ remained mostly constant across the transect (Table S3).

Spatial pattern of change in *D*_IS_:*D*_ST_ across all simulated scenarios exhibited a similar sigmoidal pattern as noted in the empirical dataset. Although the overall sigmoidal pattern of change does not differ between generation 300 and generation 1,020, the *D*_IS_:*D*_ST _values were larger near the northern limit of the hybrid zone during generation 1,020 (Figure S3). The largest increase in *D*_IS_:*D*_ST _was noted for scenarios A.ii and B.ii at generation 1,020 relative to that at 300.

## 4 DISCUSSION

Reduced genetic diversity and maladaptive gene flow from core populations limit species geographical range expansions (Gilbert et al., 2017). Using a combination of individual‐based spatial simulations and a genome‐wide empirical dataset, we examined how differences in regional population size between parental species and the hybrid zone (see Landscape set‐up under Appendix S1) along with adaptive introgression can overcome these constraints in a white pine species complex. Linking individual‐based, spatially explicit simulations with empirical analysis of genomic data has enabled us and several others (Cushman, 2014, 2015) to predict and explain the influence of complex genetic processes such as introgression and varying divergence histories on patterns of genetic diversity across the landscape. Our results demonstrate that adaptive introgression is likely facilitating northward range expansion in *Pinus strobiformis*, beyond that which could be attributed to differences in regional population size alone, as seen in our simulation study. However, we caution that conservation efforts at the species or population level should not solely rely on results from cline analysis, and that results such as ours should ideally spawn further detailed studies to aid management of interacting species.

### 4.1 Climate‐driven movement of the hybrid zone

Both direct and indirect influences of climate change can impact spatio‐temporal dynamics of hybrid zones (McQuillan & Rice, 2015). Direct influences can be brought about by climate‐mediated changes in the location of the hybrid populations. This is often apparent when hybrid zones are well established and locally adapted to their environment. Here, the warming climate could cause hybrid zone movement in the direction of the favourable optima (McQuillan & Rice, 2015; Taylor, White, et al., 2014). Indirect influences involve the modulation of species interactions through climate change, for example, shifts in species range boundaries mediated through geological events (Taylor, Curry, White, Ferretti, & Lovette, 2014; Walls, 2009). Such changes can influence the age class and ancestry composition of hybrid populations, causing them to exhibit a shift towards the more abundant species. The presence of weak isolating barriers between *P. strobiformis* and *P. flexilis*, asymmetrical niche divergence of the hybrid zone and speciation being primarily driven by extrinsic processes (Menon et al., 2018b; Moreno‐Letelier et al., 2013), can interact with changing climatic conditions to directly modulate the directionality and the rate of introgression. The long lifespan of tree species and their sessile nature also makes them prone to indirect influences of climate change over longer geological time scales, such as the Pleistocene glacial cycles. Specifically, the ancestry composition of populations within the *P. strobiformis*–*P. flexilis* hybrid zone might have been shifted towards *P. flexilis* during the glacial maxima of late Pleistocene due to range contraction and glacial refugia populations of *P. flexilis* dominating this region (Menon et al., 2018b). On the contrary, contemporary climate warming is more likely to favour the northward expansion *P. strobiformis*‐ and *P. strobiformis*‐like hybrids into the range of *P. flexilis* as noted by Shirk et al. (2018). Overall, for long‐lived tree species with weak interspecific isolating barriers, climate can impart direct and indirect influences on hybrid zone dynamics.

Combined results from geographic and genomic cline analyses support both the direct influence and the indirect influence of climate in driving hybrid zone movement and facilitating northward range expansion of *P. strobiformis*. We have demonstrated a lack of coincidence in geographic cline centre estimates between morphological and genome‐wide nuclear data, indicating a 446 km northward shift in the hybrid zone for the genomic dataset (Figure 1). Despite the fewer number of SNPs with *P. flexilis* ancestry in the hybrid zone (positive *α* values in our original BGC analysis; Menon et al., 2018b), these SNPs showed higher than expected associations with a northward shifted cline centre as compared to that of loci with *P. strobiformis* ancestry (Table 3). Such coincidence between locus‐specific ancestry and geographic centre estimate emphasises the role of hybridization in providing novel combinations of variants not present in populations representing either of the genomically pure parental species*.* Since *P. flexilis* occur in areas with lower temperatures and more frequent and prolonged freezing events compared to *P. strobiformis* (Moreno‐Letelier et al., 2013), we hypothesize that some of these directionally introgressed SNPs from *P. flexilis* might be associated with or linked to SNPs affecting freeze tolerance. These hybrid populations are poised to be an ideal source for assisted migration efforts due to their drought and freeze tolerance traits being intermediate of the two parental species (Borgman, Schoettle, & Angert, 2015; Goodrich, Waring, & Kolb, 2016).

### 4.2 Role of demographic processes

Although rare, previous studies have demonstrated hybrid zone movement in the direction opposite of that predicted by global climate change. For example, in an oak species complex, differences in relative abundances among *Quercus* species at the zone of contact have been shown to drive the genomic composition of hybrid individuals and the directionality of backcrossing (Lepais et al., 2009). In this study system, undocumented differences in relative abundance (but see Appendix S1 for proxy estimates) or ongoing introgression only from *P. flexilis* (Menon et al., 2018b) could also be driving the inferred hybrid zone movement. If true, such recent introgression at the expanding range fronts could globally elevate within‐population LD relative to the among‐population LD (Ohta, 1982), as observed at the northern range front of the hybrid zone in the empirical data herein.

**Figure 5:**
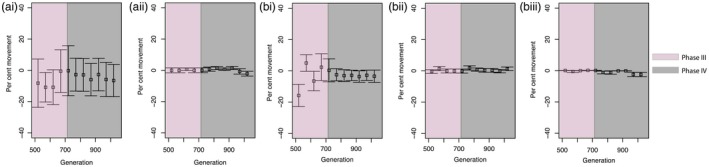
Relative percentage change in the geographic cline centre estimate across generations for all five simulated scenarios in CDMetaPOP. The change at any given generation is relative to the estimate at generation 500 and to the total spatial extent of the simulated landscape

Patterns of LD have had a long history of use to detect hybrid zone dynamics (Dasmahapatra et al., 2002; Mallet et al., 1990; Wang et al., 2011); however, the implementation of variance partitioning to distinguish between genetic drift and selection has been infrequent. A systematic increase in the values of *D*_IS_:*D*_ST _towards the northern edge of the *P. strobiformis*–*P. flexilis* hybrid zone could indicate recent colonization and ongoing northward range expansion (Wang et al., 2011), whereas lower values near the southern edge of the hybrid zone likely indicate the historical location of the hybrid zone (Latta & Mitton, 1999). Several other potential explanations also fit the noted sigmoidal pattern of *D*_IS_:*D*_ST_ (Figure 4a) uncovered in our study. First, the utilization of nearly diagnostic SNPs could create an ascertainment bias towards SNPs under extrinsic or intrinsic selection pressures, which typically tend to experience lower recombination rates. We addressed this concern (a) by generating *D*_IS_:*D*_ST_ estimates for 20 bootstrapped sets of 4,857 neutral loci each and (b) by obtaining *D*_IS_:*D*_ST _estimates across all simulated scenarios that did not incorporate any form of selection. We noticed a similar pattern of sigmoidal change for neutral loci in the empirical dataset (Figure 5b) and across all simulated scenarios (Figure S3). First, demographic processes such as serial bottlenecks following range expansions, ongoing hybridization only between *P. flexilis* and the hybrid zone and increased landscape fragmentation could have contributed towards this sigmoidal change. Second, the assignment of populations to overlapping sets could underestimate *D*_ST_ either due to similar selection pressures across the landscape defined by these populations or high levels of connectivity among populations within a set. However, the marked increase in *D*_IS _at the northern extent of the hybrid zone while *D*_ST_ remained mostly stable across the transect indicates that the second scenario is unlikely (Table S3). Third, the higher degree of landscape fragmentation and smaller populations sizes within the hybrid zone could alone increase genetic drift and cause *D*_IS _to be elevated. We addressed this possibility by comparing estimates of *D*_IS_:*D*_ST_ between generation 300 (Phase III) and generation 1,020 (end of Phase IV). The only difference between these two phases was the implementation of spatially restricted gene flow at Phase IV (Figure 2). Thus, any difference in *D*_IS_:*D*_ST _between the two points could not have resulted from population fragmentation. Although the overall sigmoidal pattern of change does not differ between generation 300 and generation 1,020, the *D*_IS_:*D*_ST _values were larger near the northern limit of the hybrid zone during generation 1,020 (Figure S3), indicating the role of spatially biased introgression in driving the noted northward hybrid zone movement. This combined effect of range expansion, spatially restricted hybridization and population fragmentation is likely also driving the overall higher estimates of *D*_IS_:*D*_ST _in the neutral empirical dataset in relation to the nearly diagnostic SNPs (Figure 4). Overall, while the *D*_IS_:*D*_ST _approach and contingency analysis show selection to have played an important role, it is unlikely to be the sole player in the observed northward movement of the hybrid zone.

Across both models of divergence in our simulated dataset, we noticed certain time points with steep shifts in the geographic cline centre (scenario A.i and B.i, Figure 5). A consistent maintenance of the shifted centre across generations is specifically evident during Phase IV of A.i and B.i. This observed pattern agrees with spatio‐temporal changes in range margins of the focal species being caused by asymmetrical patterns of gene flow, driven by differences in regional population sizes. On the contrary, simulation scenarios representing the contemporary pattern of gene flow in our study system (i.e., restricted between *P. flexilis* and the hybrid zone) exhibited a stable geographic cline centre across time (Scenario A.ii and B.ii, Figure 5). Scenarios with the most drastic shift in the geographic cline centre were also associated with higher variance across replicate runs, likely reflecting the stochastic nature of the simulations introduced by recurring high levels of gene flow and rapid population composition turnover across generations. Overall, our results indicate that it is not possible to distinguish between Model A and Model B solely based on spatio‐temporal shifts in the geographic cline centre as these were predominantly driven by recent patterns of gene flow.

### 4.3 Role of intrinsic processes

In addition to differences in regional population sizes, the asymmetric pattern of introgression could result from intrinsic selection pressures such as the formation of co‐adapted gene complexes (Barton & Hewitt, 1985). For example, in the hybrid zone between Townsend and Hermit Warblers, a downward latitudinal shift in the hybrid zone has been attributed to fitness differences and competitive superiority of Townsend Warbler, which is a more northerly species (Krosby & Rohwer, 2009). In line with studies across various systems demonstrating asymmetric patterns of introgression (Souissi et al., 2018; Suarez‐Gonzalez, Hefer, Lexer, Cronk, & Douglas, 2018; Wellenreuther et al., 2018), our study indicates that the majority of highly introgressed SNPs in the hybrid zone are representative of *P. strobiformis* (negative *α* in BGC and skewed distribution of hybrid index). We suggest that this pattern of genome‐wide asymmetry is likely a consequence of the initial colonization of discontinuous populations of the southwestern United States and northern Mexico occurring from pure *P. strobiformis* populations only and asymmetric differences in fitness among hybrid individuals, as seen in the warbler hybrid zone noted above. Despite the noted dominance of negative *α* outliers (*P. strobiformis* ancestry), the results from our contingency analysis demonstrated a strong influence of introgression from *P. flexilis* in facilitating northward hybrid zone movement (Table 3). Comparisons between the empirical and simulated datasets demonstrated that although differences in regional population size of each species and intrinsic selection pressures can drive spatio‐temporal dynamics of the hybrid zone under certain scenarios of gene flow, the magnitude of movement observed in our study is unlikely to have occurred without invoking adaptive introgression from *P. flexilis*.

### 4.4 Implications for forest management

Overall, our results are consistent with a northward expansion of the hybrid zone being driven by the formation of novel gene combinations created through introgression from both *P. strobiformis* and *P. flexilis*. A similar pattern has been noted in the hybrid zone between *Populus balsamifera* and *P. trichocarpa*, where northward expansion of *P. trichocarpa* is facilitated by introgression of cold‐adapted alleles from pure *P. balsamifera* and pest‐resistant alleles from pure *P. trichocarpa* (Suarez‐Gonzalez et al., 2018). Such novel combinations are specifically useful for tree species due to their long generation time and sessile nature, making them more susceptible to rapidly changing climatic conditions. Ongoing research related to the ability of each species to adapt or migrate under climate change, such as common garden studies, coupled with investigations to identify trees and populations with genetic resistance to white pine blister rust (caused by *Cronartium ribicola*), can be synthesised with the current study to provide recommendations for forest managers (sensu Schoettle, Burns, Cleaver, & Connor, 2018; Sniezko, Kegley, & Danchok, 2008; Waring et al., 2017). For example, conservation of parental species versus hybrid populations within the expanding front will be determined by differences in their ability to withstand the dual pressure of freezing temperatures and white pine blister rust. However, coincidence of both biotic and abiotic stress tolerance traits may be problematic considering that they may be interrelated (Vogan & Schoettle, 2015, 2016). Documenting this trade‐off across populations and among hybrid versus parental species will be important for management efforts given the projected northward range expansion of the hybrid zone and continued spread of *C. ribicola* (Schwandt, Lockman, Kliejunas, & Muir, 2010). Preliminary data from common garden trials screening for genetic resistance to *C. ribicola*, however, suggest higher quantitative resistance in hybrid populations in comparison with pure *P. flexilis* (*Pers. Comm.* Sniezko RA and Schoettle AW). If this pattern holds true, the putative combination of greater freeze tolerance (in comparison with pure *P. strobiformis*) and higher resistance to white pine blister rust (in comparison with pure *P. flexilis*) in the hybrid populations will make them an ideal source for assisted migration and germplasm conservation efforts. Future and ongoing research across several common gardens will be able to address this hypothesis in detail.

Managers considering outplanting of seedlings, a key strategy for resilience of both species (Schoettle, Jacobi, Waring, & Burns, 2019), should include the expanded hybrid zone identified herein as this might contain novel adaptive trait combinations, while not neglecting southern or isolated populations that may also contain important adaptive traits related to drought stress (Hampe & Petit, 2005). For example, the southern US and Mexican populations may be able to withstand higher drought stress that is projected to occur under the current climate scenario for western North America and hence could be a sustainable source of pure *P. strobiformis*. Approved seed zones further complicate efforts to select the best planting stock for future conditions since these typically do not incorporate information about hybridization or shifting climate niches. Communication between researchers and managers will be important as new research results become available, to enable managers to make the best choices under existing management and budgetary frameworks.

Our findings present a valuable case study showing that introgression has contributed to increasing genetic diversity in marginal populations and facilitated adaptive evolution in a forest tree hybrid zone. As emphasized since the late 1950s by Stebbins (1959) and recently reviewed by Janes and Hamilton (2017), hybridization can provide genetic resources to assist rapid adaptive evolution and range shifts in species such as *P. strobiformis* and *P. flexilis*, which are confronting the dual threats of rapidly changing climate and the invasive tree disease, white pine blister rust. Documenting genetic diversity across the range of the entire species and of the principal hybridizing species is useful for identifying populations requiring the most intervention and for identifying novel seed sources for future reforestation and assisted migration efforts (Aitken & Whitlock, 2013). Thus, whether long‐lived species such as forest trees will adapt, move, or exhibit plastic responses in in the face of rapidly changing climate (Aitken, Yeaman, Holliday, Wang, & Curtis‐McLane, 2008) will depend on the availability of standing genetic variation, which is constantly modulated by the dynamic nature of landscapes resulting in new and altered species interactions through time.

## CONFLICT OF INTEREST

None declared.

## AUTHOR CONTRIBUTIONS

The study was conceived by MM, AJE & EL. Funding was procured by KMW, SC, LFR, AJE & CW. Field sampling and curation of morphological data were conducted by AWS, CW & ALS. EL set up the simulations in CDMetaPOP. JCB conducted the genomic cline analysis in BGC. MM analysed the data, ran the simulations and wrote the manuscript. All authors edited the manuscript and have approved this version for submission.

## DATA ACCESSIBILITY

Genomic data used in this study are publicly available through Dryad at https://doi.org/10.5061/dryad.f6r55. Scripts used for running simulations and analysing the data are publicly available via github (https://github.com/mitramenon/Hybrid-zone-mov). Simulation data sets from CDMetaPOP are available through Dryad at https://doi.org/10.5061/dryad.qp59ck8.

## References

[eva12795-bib-0001] Abbott, R. J. , Barton, N. H. , & Good, J. M. (2016). Genomics of hybridization and its evolutionary consequences. Molecular Ecology, 25, 2325–2332. 10.1111/mec.13685 27145128

[eva12795-bib-0002] Aitken, S. N. , & Whitlock, M. C. (2013). Assisted gene flow to facilitate local adaptation to climate change. Annual Review of Ecology Evolution and Systematics, 44, 367–388. 10.1146/annurev-ecolsys-110512-135747

[eva12795-bib-0003] Aitken, S. N. , Yeaman, S. , Holliday, J. A. , Wang, T. , & Curtis‐McLane, S. (2008). Adaptation, migration or extirpation: Climate change outcomes for tree populations. Evolutionary Applications, 1, 95–111. 10.1111/j.1752-4571.2007.00013.x 25567494 PMC3352395

[eva12795-bib-0004] Arntzen, J. W. , de Vries, W. , Canestrelli, D. , & Martínez‐Solano, I. (2017). Hybrid zone formation and contrasting outcomes of secondary contact over transects in common toads. Molecular Ecology, 26(20), 5663–5675. 10.1111/mec.14273 28752635

[eva12795-bib-0005] Barton, N. H. , & Gale, K. S. (1993). Genetic analysis of hybrid zones In Harrison R. G. (Ed.), Hybrid zones and the evolutionary process (pp. 13–42). Oxford, UK: Oxford University Press.

[eva12795-bib-0006] Barton, N. H. , & Hewitt, G. M. (1985). Analysis of hybrid zones. Annual Review of Ecology and Systematics, 16, 113–148. 10.1146/annurev.es.16.110185.000553

[eva12795-bib-0007] Beissinger, T. M. , Gholami, M. E. , Weigend, S. , Weigend, A. , de Leon, N. , Gianola, D. , & Simianer, H. (2015). Using the variability of linkage disequilibrium between subpopulations to infer sweeps and epistatic selection in a diverse panel of chickens. Heredity, 116, 58–166. 10.1038/hdy.2015.8 PMC4806882 26350629

[eva12795-bib-0008] Bisbee, J. (2014). Cone morphology of the *Pinus ayacahuite‐flexilis* complex of the southwestern United States and Mexico. Bulletin of the Cupressus Conservation Project, 3, 3–33.

[eva12795-bib-0009] Borgman, E. M. , Schoettle, A. W. , & Angert, A. L. (2015). Assessing the potential for maladaptation during active management of limber pine populations: A common garden study detects genetic differentiation in response to soil moisture in the Southern Rocky Mountains. Canadian Journal of Forest Research, 45, 496–505. 10.1139/cjfr-2014-0399

[eva12795-bib-0010] Britch, S. C. , Cain, M. L. , & Howard, D. J. (2008). Spatio‐temporal dynamics of the *Allonemobius fasciatus*‐*A. Socius* mosaic hybrid zone: A 14‐year perspective. Molecular Ecology, 10(3), 627–638. 10.1046/j.1365-294x.2001.01215.x 11298974

[eva12795-bib-0011] Brown, J. H. (1984). On the relationship between abundance and distribution of species. American Naturalist, 124, 255–279. 10.1086/284267

[eva12795-bib-0012] Buggs, R. (2007). Empirical study of hybrid zone movement. Heredity, 99(3), 301–312. 10.1038/sj.hdy.6800997 17611495

[eva12795-bib-0013] Critchfield, W. B. (1986). Hybridization and classification of the white pines (*Pinus* section *Strobus*). Taxon, 35, 647–656. 10.2307/1221606

[eva12795-bib-0014] Currat, M. , Ruedi, M. , Petit, R. J. , & Excoffier, L. (2008). The hidden side of invasions: Massive introgression by local genes. Evolution, 62, 1908–1920. 10.1111/j.1558-5646.2008.00413.x 18452573

[eva12795-bib-0015] Cushman, S. A. (2014). Grand challenges in evolutionary and population genetics: The importance of integrating epigenetics, genomics, modeling, and experimentation. Frontiers in Genetics, 5, 197 10.3389/fgene.2014.00197 25071827 PMC4085548

[eva12795-bib-0016] Cushman, S. A. (2015). Pushing the envelope in genetic analysis of species invasion. Molecular Ecology, 24, 259–262. 10.1111/mec.13043 25594583

[eva12795-bib-0017] Dasmahapatra, K. K. , Blum, J. M. , Aiello, A. , Hackwell, S. , Davies, N. , Bermingham, E. P. , & Mallet, J. (2002). Inferences from a rapidly moving hybrid zone. Evolution, 56(4), 741–753. 10.1111/j.0014-3820.2002.tb01385.x 12038532

[eva12795-bib-0018] de Lafontaine, G. , Prunier, J. , Gérardi, S. , & Bousquet, J. (2015). Tracking the progression of speciation: variable patterns of introgression across the genome provide insights on the species delimitation between progenitor-derivative spruces (*Picea mariana × P. rubens*). Molecular Ecology, 24(20), 5229–5247. 26346701 10.1111/mec.13377

[eva12795-bib-0019] Derryberry, E. P. , Derryberry, G. E. , Maley, J. M. , & Brumfield, R. T. (2013). hzar: Hybrid zone analysis using an R software package. Molecular Ecology Resources, 14, 652–663. 10.1111/1755-0998.12209 24373504

[eva12795-bib-0020] Eckert, C. , Samis, K. , & Lougheed, S. (2008). Genetic variation across species’ geographical ranges: The central– marginal hypothesis and beyond. Molecular Ecology, 17(5), 1170–1188. 10.1111/j.1365-294X.2007.03659.x 18302683

[eva12795-bib-0021] Engler, J. O. , Rödder, D. , Elle, O. , Hochkirch, A. , & Secondi, J. (2013). Species distribution models contribute to determine the effect of climate and interspecific interactions in moving hybrid zones. Journal of Evolutionary Biology, 26(11), 2487–2496. https://doi.org.10.1111/jeb.12244 24016292 10.1111/jeb.12244

[eva12795-bib-0022] Fitzpatrick, B. M. , Johnson, J. R. , Kump, D. K. , Smith, J. J. , Voss, S. R. , & Shaffer, H. B. (2010). Rapid spread of invasive genes into a threatened native species. Proceedings of the National Academy of Sciences USA, 107(8), 3606–3610. 10.1073/pnas.0911802107 PMC2840512 20133596

[eva12795-bib-0023] Frankis, M. (2009). The high altitude white pines of Mexico and the adjacent SW USA (*Pinus* L. subgenus *Strobus* Lemmon, Pinaceae). International Dendrology Society Yearbook, 2008, 63–72.

[eva12795-bib-0024] Gay, L. , Crochet, P. A. , Bell, D. A. , & Lenormand, T. (2008). Comparing clines on molecular and phenotypic traits in hybrid zones: A window on tension zone models. Evolution, 62(11), 2789–2806. 10.1111/j.1558-5646.2008.00491.x 18752618

[eva12795-bib-0025] Gilbert, K. J. , Sharp, N. P. , Angert, A. L. , Conte, G. L. , Draghi, J. A. , Guillaume, F. , … Whitlock, M. C. (2017). Local adaptation interacts with expansion load during range expansion: Maladaptation reduces expansion load. American Naturalist., 189(4), 368–380. 10.1086/690673 28350500

[eva12795-bib-0026] Gompert, Z. , & Buerkle, C. A. (2012). bgc: Software for Bayesian estimation of genomic clines. Molecular Ecology Resources, 12(6), 1168–1176. 10.1111/1755-0998.12009 22978657

[eva12795-bib-0027] Gompert, Z. , Parchman, T. L. , & Buerkle, C. A. (2012). Genomics of isolation in hybrids. Philosophical Transactions of the Royal Society B: Biological Sciences, 367, 439–450. 10.1098/rstb.2011.0196 PMC3233709 22201173

[eva12795-bib-0028] Goodrich, B. A. , Waring, K. M. , & Flores‐Renteria, L. (2018). *Pinus strobiformis* gene conservation and genecology In Schoettle A. W., Sniezko R. A., & Kliejunas J. T. (Eds.), Proceedings of the IUFRO joint conference: Genetics of five‐needle pines, rusts of forest trees, and Strobusphere; 2014 June 15–20; Fort Collins, CO. Proc. RMRS‐P‐76 (pp. 21–29). Fort Collins, CO: U.S. Department of Agriculture, Forest Service, Rocky Mountain Research Station. P: 245.

[eva12795-bib-0029] Goodrich, B. A. , Waring, K. M. , & Kolb, T. E. (2016). Genetic variation in *Pinus strobiformis* growth and drought tolerance from southwestern US populations. Tree Physiology, 36, 1219–1235. 10.1093/treephys/tpw052 27344065

[eva12795-bib-0030] Goudet, J. (2005). hierfstat, a package for R to compute and test hierarchical F‐statistics. Molecular Ecology Notes, 5, 184–186. 10.1111/j.1471-8286.2004.00828.x

[eva12795-bib-0031] Hamilton, J. A. , & Miller, J. M. (2016). Adaptive introgression as a resource for management and genetic conservation in a changing climate. Conservation Biology, 30(1), 33–41. 10.1111/cobi.12574 26096581

[eva12795-bib-0032] Hampe, A. , & Petit, R. J. (2005). Conserving biodiversity under climate change: The rear edge matters. Ecology Letters, 8(5), 461–467. 10.1111/j.1461-0248.2005.00739.x 21352449

[eva12795-bib-0033] Hijmans, R. J. (2017). *geosphere: Spherical trigonometry* . R package version 1.5‐7.

[eva12795-bib-0034] Ikeda, D. H. , Max, T. L. , Allan, G. J. , Lau, M. K. , Shuster, S. M. , & Whitham, T. G. (2017). Genetically informed ecological niche models improve climate change prediction. Global Change Biology, 23(1), 164–176. 10.1111/gcb.13470 27543682

[eva12795-bib-0035] Jagoda, E. , Lawson, D. J. , Wall, J. D. , Lambert, D. , Muller, C. , Westaway, M. , … Pagani, L. (2017). Disentangling immediate adaptive introgression from selection on standing introgressed variation in humans. Molecular Biology and Evolution, 35(3), 623–630. 10.1093/molbev/msx314 PMC5850494 29220488

[eva12795-bib-0036] Janes, J. K. , & Hamilton, J. A. (2017). Mixing it up: The role of hybridization in forest management and conservation under climate change. Forests, 8(7), 237 10.3390/f8070237

[eva12795-bib-0037] Krosby, M. , & Rohwer, S. (2009). A 2000 km genetic wake yields evidence for northern glacial refugia and hybrid zone movement in a pair of songbirds. Proceedings of the Royal Society B: Biological Sciences, 276(1657), 615–621. 10.1098/rspb.2008.1310 PMC2660942 18986973

[eva12795-bib-0038] Lamichhaney, S. , Han, F. , Webster, M. T. , Andersson, L. , Grant, B. R. , & Grant, P. R. (2018). Rapid hybrid speciation in Darwin’s finches. Science, 359(6372), 224–228. 10.1126/science.aao4593 29170277

[eva12795-bib-0039] Landguth, E. L. , Bearlin, A. , Day, C. C. , & Dunham, J. (2016). CDMetaPOP: An individual‐based, eco‐evolutionary model for spatially explicit simulation of landscape demogenetics. Methods in Ecology and Evolution, 8, 4–11. 10.1111/2041-210X.12608

[eva12795-bib-0040] Larson, M. , & Moir, W. H. (1987). Forest and woodland habitat types (plant associations) of northern New Mexico and northern Arizona (2nd ed., p. 90). Albuquerque, NM: U.S. Department of Agriculture, Forest Service, Southwestern Region.

[eva12795-bib-0041] Latta, R. G. , & Mitton, J. B. (1999). Historical separation and present gene flow through a zone of secondary contact in ponderosa pine. Evolution, 53, 769–776. 28565634 10.1111/j.1558-5646.1999.tb05371.x

[eva12795-bib-0042] Lawton, J. H. (1993). Range, population abundance and conservation. Trends in Ecology and Letters, 8, 409–413. 10.1016/0169-5347(93)90043-O 21236213

[eva12795-bib-0043] Leal‐Saenz, A. , Menon, M. , Waring, K. , Sniezko, R. , Hernández‐Díaz, J. C. , López‐Sanchez, C. A. , … Wehenkel, C. (2018). Morphological differences in *Pinus strobiformis* across latitudinal and elevational gradients. Frontier in Plant Science, in review. 10.3389/fpls.2020.559697 PMC7642095 33193485

[eva12795-bib-0044] Lepais, O. , Petit, R. J. , Guichoux, E. , Lavabre, J. E. , Alberto, F. , Kremer, A. , & Gerber, S. (2009). Species relative abundance and direction of introgression in oaks. Molecular Ecology, 18(10), 2228–2242. 10.1111/j.1365-294X.2009.04137.x 19302359

[eva12795-bib-0045] Looney, C. E. , & Waring, K. M. (2013). *Pinus strobiformis* (southwestern white pine) stand dynamics, regeneration, and disturbance ecology: A review. Forest Ecology and Management., 287, 90–102. 10.1016/j.foreco.2012.09.020

[eva12795-bib-0046] Mallet, J. , Barton, N. , Lamas, G. , Santisteban, J. , Muedas, M. , & Eeley, H. (1990). Estimates of selection and gene flow from measures of cline width and linkage disequilibrium in heliconius hybrid zones. Genetics, 124(4), 921–936. 2323556 10.1093/genetics/124.4.921 PMC1203983

[eva12795-bib-0047] Malone, S. L. , Schoettle, A. W. , & Coop, J. D. (2018). The future of subalpine forests in the Southern Rocky Mountains: Trajectories for *Pinus aristata* genetic lineages. PLoS ONE, 13(3), e0193481 10.1371/journal.pone.0193481 29554097 PMC5858753

[eva12795-bib-0048] Martin, L. , & Cruzan, M. (1999). Patterns of hybridization in the Piriqueta caroliniana complex in central Florida: Evidence for an expanding hybrid zone. Evolution, 53(4), 1037–1049. 10.1111/j.1558-5646.1999.tb04519.x 28565512

[eva12795-bib-0049] McQuillan, M. A. , & Rice, A. M. (2015). Differential effects of climate and species interactions on range limits at a hybrid zone: Potential direct and indirect impacts of climate change. Ecology and Evolution, 5(21), 5120–5137. 10.1002/ece3.1774 26640687 PMC4662315

[eva12795-bib-0050] Meier, J. I. , Marques, D. A. , Mwaiko, S. , Wagner, C. E. , Excoffier, L. , & Seehausen, O. (2017). Ancient hybridization fuels rapid cichlid fish adaptive radiations. Nature Communications, 8, 14363 10.1038/ncomms14363 PMC5309898 28186104

[eva12795-bib-0051] Menon, M. , Bagley, J. C. , Friedline, C. J. , Whipple, A. V. , Schoettle, A. W. , Lael‐Saenz, A. , … Eckert, A. J. (2018a). Genotype file. Dryad Digital Repository, 10.5061/dryad.f6r55

[eva12795-bib-0052] Menon, M. , Bagley, J. C. , Friedline, C. J. , Whipple, A. V. , Schoettle, A. W. , Leal‐Sàenz, A. , … Eckert, A. J. (2018b). The role of hybridization during ecological divergence of southwestern white pine (*Pinus strobiformis*) and limber pine (*P. flexilis*). Molecular Ecology, 27, 1245–1260. 10.1111/mec.14505 29411444

[eva12795-bib-0053] Menon, M. , Landguth, E. , Leal‐Saenz, A. , Bagley, J. , Schoettle, A. W. , Wehenkel, C. , … Eckert, A. J. (2019). Simulations file. Dryad Digital Repository, 10.5061/dryad.qp59ck8

[eva12795-bib-0054] Moltke, I. , & Albrechtsen, A. (2014). RelateAdmix: A software tool for estimating relatedness between admixed individuals. Bioinformatics, 30(7), 1027–1028. 10.1093/bioinformatics/btt652 24215025

[eva12795-bib-0055] Moreno‐Letelier, A. , Ortíz‐Medrano, A. , & Piñero, D. (2013). Niche divergence versus neutral processes: Combined environmental and genetic analyses identify contrasting patterns of differentiation in recently diverged pine species. PLoS ONE, 8, e78228 10.1371/journal.pone.0078228 24205167 PMC3812143

[eva12795-bib-0056] Mosseler, A. , Major, J. E. , Simpson, J. D. , Daigle, B. , Lange, K. , Park, Y. S. , … Rajora, O. P. (2000). Indicators of population viability in red spruce, *Picea rubens*. I. Reproductive traits and fecundity. Canadian Journal of Botany, 78(7), 928–940. 10.1139/b00-065

[eva12795-bib-0057] Ohta, T. (1982). Linkage disequilibrium with the island model. Genetics, 101(1), 139–155. 17246079 10.1093/genetics/101.1.139 PMC1201847

[eva12795-bib-0058] Parchman, T. L. , Gompert, Z. , Mudge, J. , Schilkey, F. D. , Benkman, C. W. , & Buerkle, C. A. (2012). Genome‐wide association genetics of an adaptive trait in lodgepole pine: Association mapping of serotiny. Molecular Ecology, 21, 2991–3005. 10.1111/j.1365-294X.2012.05513.x 22404645

[eva12795-bib-0059] Pfenning, K. S. , Kelly, A. L. , & Pierce, A. A. (2016). Hybridization as a facilitator of species range expansion. Proceedings of the Royal Society B: Biological Sciences, 283, 20161329. 10.1098/rspb.2016.1329 PMC5046898 27683368

[eva12795-bib-0060] Pironon, S. , Papuga, G. , Villellas, J. , Angert, A. L. , García, M. B. , & Thompson, J. D. (2017). Geographic variation in genetic and demographic performance: New insights from an old biogeographical paradigm. Biological Reviews of the Cambridge Philosophical Society, 92, 1877–1909. 10.1111/brv.12313 27891813

[eva12795-bib-0061] Pollock, L. J. , Tingley, R. , Morris, W. K. , Golding, N. , O'Hara, R. B. , Parris, K. M. , … McCarthy, M. A. (2014). Understanding co‐occurrence by modelling species simultaneously with a Joint Species Distribution Model (JSDM). Methods in Ecology and Evolution, 5(5), 397–406. 10.1111/2041-210X.12180

[eva12795-bib-0062] Puritz, J. B. , Hollenbeck, C. M. , & Gold, J. R. (2014). dDocent : A RADseq, variant‐calling pipeline designed for population genomics of non‐model organisms. Peerj, 2, e431 10.7717/peerj.431 24949246 PMC4060032

[eva12795-bib-0063] R Core Team (2017). R: A language and environment for statistical computing. Vienna, Austria: Computing. R Foundation for Statistical Computing.

[eva12795-bib-0064] Raj, A. , Stephens, M. , & Pritchard, J. K. (2014). fastSTRUCTURE: Variational inference of population structure in large SNP data sets. Genetics, 197(2), 573–589. 10.1534/genetics.114.164350 24700103 PMC4063916

[eva12795-bib-0065] Rieseberg, L. H. , Kim, S. C. , Randell, R. A. , Whitney, K. D. , Gross, B. L. , Lexer, C. , & Clay, K. (2007). Hybridization and the colonization of novel habitats by annual sunflowers. Genetics, 129(2), 149–165. 10.1007/s10709-006-9011-y PMC2442915 16955330

[eva12795-bib-0066] Rohwer, S. , Bermingham, E. , & Wood, C. (2001). Plumage and mitochondrial DNA haplotype variation across a moving hybrid zone. Evolution, 55(2), 405–422. 10.1111/j.0014-3820.2001.tb01303.x 11308096

[eva12795-bib-0067] Schoettle, A. W. , Burns, K. S. , Cleaver, C. M. , & Connor, J. J. (2018). Proactive limber pine conservation strategy for the Greater Rocky Mountain National Park Area. General Technical Report RMRS‐GTR‐379 (81 pp). Fort Collins, CO: U.S. Department of Agriculture, Forest Service, Rocky Mountain Research Station.

[eva12795-bib-0068] Schoettle, A. W. , Jacobi, W. R. , Waring, K. M. , & Burns, K. S. (2019). Regeneration for resilience framework to support regeneration decisions for species with populations at risk of extirpation by white pine blister rust. New Forests, 50, 89–114. 10.1007/s11056-108-9679-8

[eva12795-bib-0069] Schwandt, J. W. , Lockman, I. B. , Kliejunas, J. T. , & Muir, J. A. (2010). Current health issues and management strategies for white pines in the western United States and Canada. Forest Pathology, 40(3), 226–250. 10.1111/j.1439-0329.2010.00656.x

[eva12795-bib-0070] Sexton, J. P. , McIntyre, P. J. , Angert, A. L. , & Rice, K. J. (2009). Evolution and ecology of species range limits. Annual Review of Ecology Evolution and Systematics, 40, 415–436. 10.1146/annurev.ecolsys.110308.120317

[eva12795-bib-0071] Sheth, S. N. , & Angert, A. L. (2018). Demographic compensation does not rescue populations at a trailing range edge. Proceedings of the National Academy of Sciences USA, 115(10), 2413–2418. 10.1073/pnas.1715899115 PMC5878003 29463711

[eva12795-bib-0072] Shirk, A. J. , Cushman, S. A. , Wehenkel, C. A. , Leal‐Sáenz, A. , Toney, C. , & Lopez‐Sanchez, C. A. (2018). Southwestern white pine (*Pinus strobiformis*) species distribution models predict large range shift and contraction due to climate change. Forest Ecology & Management, 411, 176–186. 10.1016/j.foreco.2018.01.025

[eva12795-bib-0073] Sniezko, R. A. , Kegley, A. J. , & Danchok, R. (2008). White pine blister rust resistance in North American, Asian, and European species – Results from artificial inoculation trials in Oregon. Annals of Forest Research, 51, 53–66.

[eva12795-bib-0074] Souissi, A. , Bonhomme, F. , Manchado, M. , Sfar, L. B. , & Gagnaire, P. A. (2018). Genomic and geographic footprints of differential introgression between two divergent fish species (*Solea* spp.). Heredity, 121, 579–593. 10.1038/s41437-018-0079-9 29713088 PMC6221876

[eva12795-bib-0075] Stebbins, G. L. (1959). The role of hybridization in evolution. Proceedings of the American Philosophical Society, 103, 231–251.

[eva12795-bib-0076] Steinhoff, R. J. , & Andresen, J. W. (1971). Geographic variation in *Pinus flexilis* and *Pinus strobiformis* and its bearing on their taxonomic status. Silvae Genet, 20, 159–167.

[eva12795-bib-0077] Stelkens, R. B. , Brockhurst, M. A. , Hurst, G. D. , & Greig, D. (2014). Hybridization facilitates evolutionary rescue. Evolutionary Applications, 7(10), 1209–1217. 10.1111/eva.12214 25558281 PMC4275092

[eva12795-bib-0078] Suarez‐Gonzalez, A. , Hefer, C. A. , Lexer, C. , Cronk, Q. C. B. , & Douglas, C. J. (2018). Scale and direction of adaptive introgression between black cottonwood (*Populus trichocarpa*) and balsam poplar (*P. balsamifera*). Molecular Ecology, 27(7), 1667–1680. 10.1111/mec.14561 29575353

[eva12795-bib-0079] Taylor, S. A. , Curry, R. L. , White, T. A. , Ferretti, V. , & Lovette, I. (2014). Spatiotemporally consistent genomic signatures of reproductive isolation in a moving hybrid zone. Evolution, 68(11), 3066–3081. 10.1111/evo.12510 25138643

[eva12795-bib-0080] Taylor, S. A. , White, T. A. , Hochachka, W. M. , Ferretti, V. , Curry, R. L. , & Lovette, I. (2014). Climate‐mediated movement of an avian hybrid zone. Current Biology, 24(6), 671–676. 10.1016/j.cub.2014.01.069 24613306

[eva12795-bib-0081] Todesco, M. , Pascual, M. A. , Owens, G. L. , Ostevik, K. L. , Moyers, B. T. , Hübner, S. , … Rieseberg, L. H. (2016). Hybridization and extinction. Evolutionary Application, 9(7), 892–908. 10.1111/eva.12367 PMC4947151 27468307

[eva12795-bib-0082] Tomback, D. F. , Samano, S. , Pruett, E. L. , & Schoettle, A. W. (2011). Seed dispersal in limber and southwestern white pine: comparing core and peripheral populations In The future of high‐elevation, five‐needle white pines in Western North America: Proceedings of the high five symposium. Proceedings RMRS‐P‐63 (pp. 69–71). Fort Collins, CO: US Department of Agriculture, Forest Service, Rocky Mountain Research Station.

[eva12795-bib-0083] van Riemsdijk, I. , Butlin, R. K. , Wielstra, B. , & Arntzen, J. W. (2018). Testing an hypothesis of hybrid zone movement for toads in France. Molecular Ecology, 28, 1070–1083. 10.1111/mec.15005 30609055

[eva12795-bib-0084] Vogan, P. J. , & Schoettle, A. W. (2015). Selection for resistance to white pine blister rust affects the abiotic stress tolerances of limber pine. Forest Ecology and Management, 344, 110–119. 10.1016/j.foreco.2015.01.029

[eva12795-bib-0085] Vogan, P. J. , & Schoettle, A. W. (2016). Carbon costs of constitutive and expressed resistance to a non‐native pathogen in limber pine. PLoS ONE, 11(10), e0162913 10.1371/journal.pone.0162913 27706249 PMC5051957

[eva12795-bib-0086] Walls, S. W. (2009). The role of climate in the dynamics of a hybrid zone in Appalachian salamanders. Global Change Biology, 15(8), 1903–1910. 10.1111/j.1365-2486.2009.01867.x

[eva12795-bib-0087] Walsh, J. , Shriver, W. , Olsen, B. , & Kovach, A. I. (2016). Differential introgression and the maintenance of species boundaries in an advanced generation avian hybrid zone. BMC Evolutionary Biology, 16(1), 16–65. 10.1186/s12862-016-0635-y 27000833 PMC4802838

[eva12795-bib-0088] Wang, L. , Luzynski, K. , Pool, J. E. , JanouŠek, V. , DufkovÚ, P. , VyskoČilovÚ, M. M. , … Tucker, P. K. (2011). Measures of linkage disequilibrium among neighbouring SNPs indicate asymmetries across the house mouse hybrid zone. Molecular Ecology, 20(14), 2985–3000. 10.1111/j.1365-294X.2011.05148 21668551

[eva12795-bib-0089] Waring, K. , Cushman, S. , Eckert, A. , Flores Renteria, L. , Sniezko, R. , Still, S. , … Moler, E. (2017). *Blending ecology and evolution using emerging technologies to determine species distributions with a non‐native pathogen in a changing climate* . Forest Regeneration in Changing Climates, July 11–13, 2017, Corvallis, OR.

[eva12795-bib-0090] Wellenreuther, M. , Muñoz, J. , Chávez‐Ríos, J. R. , Hansson, B. , Cordero‐Rivera, A. , & Sánchez‐Guillén, R. A. (2018). Molecular and ecological signatures of an expanding hybrid zone. Ecology and Evolution, 8, 4793–4806. 10.1002/ece3.4024 29876058 PMC5980427

[eva12795-bib-0091] Wielstra, B. , Burke, T. , Butlin, R. K. , Avcı, A. , Üzüm, N. , Bozkurt, E. , … Arntzen, J. W. (2017). A genomic footprint of hybrid zone movement in crested newts. Evolution Letters, 1(2), 93–101. 30283642 10.1002/evl3.9 PMC6121819

[eva12795-bib-0092] Williams, C. G. (2010). Long‐distance pine pollen still germinates after meso‐scale dispersal. American Journal of Botany, 97(5), 846–855. 10.3732/ajb.0900255 21622450

[eva12795-bib-0093] Windmuller‐Campione, M. A. , & Long, J. N. (2016). Limber pine (*Pinus flexilis* James), a flexible generalist of forest communities in the Intermountain West. PLoS ONE, 11(8), e0160324 10.1371/journal.pone.0160324 27575596 PMC5004877

