# Supplementary figures and images for: Tracing the footprints of a moving hybrid zone under a demographic history of speciation with gene flow
Source: Evol Appl. 2019 Apr 29;13(1):195–209. doi: 10.1111/eva.12795 (PMC6935588; doi:10.1111/eva.12795)

Latitude

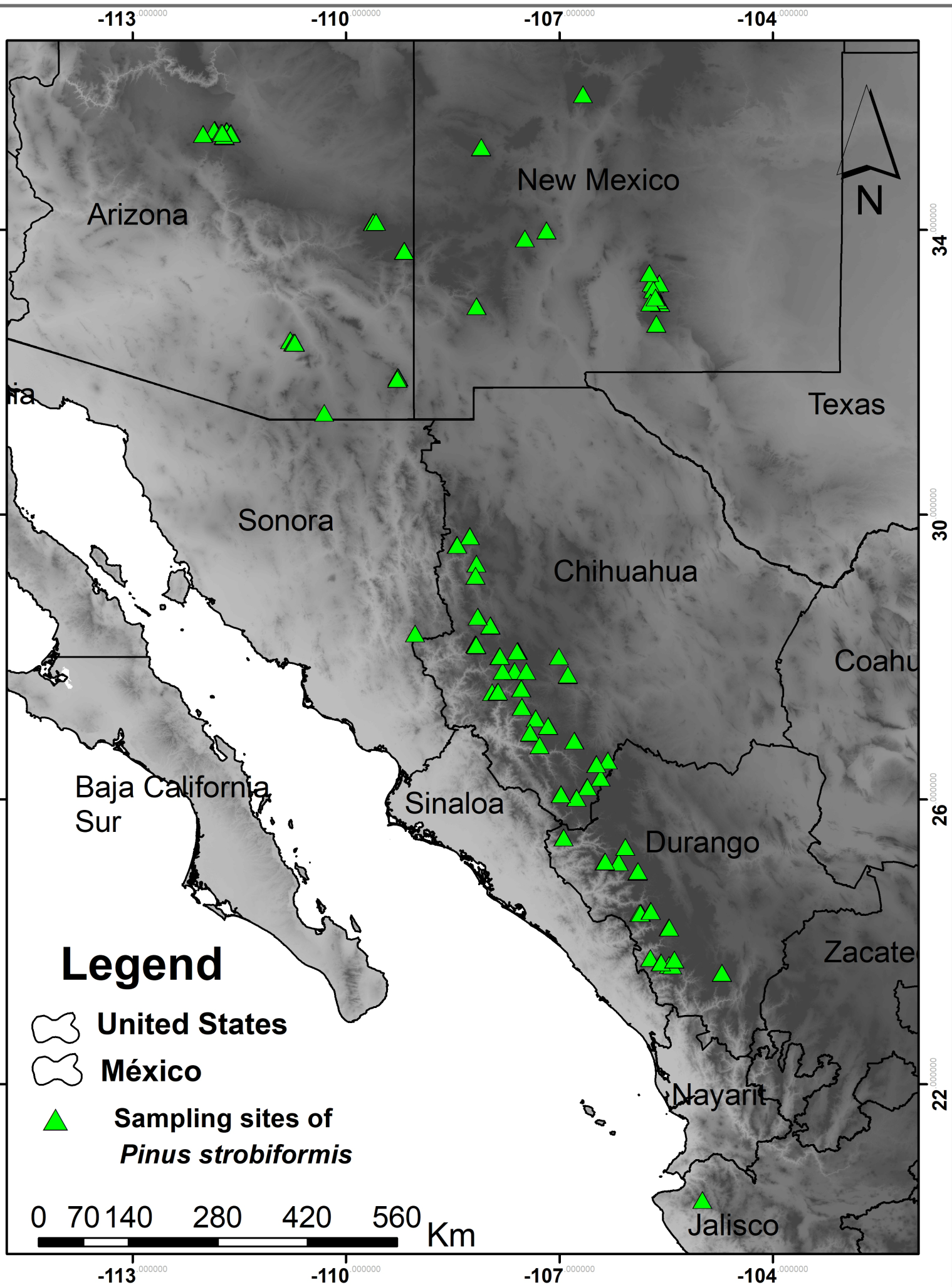

Longitude

Supplement: Supplementary file 1 [file EVA-13-195-s001.pdf]

**Ai**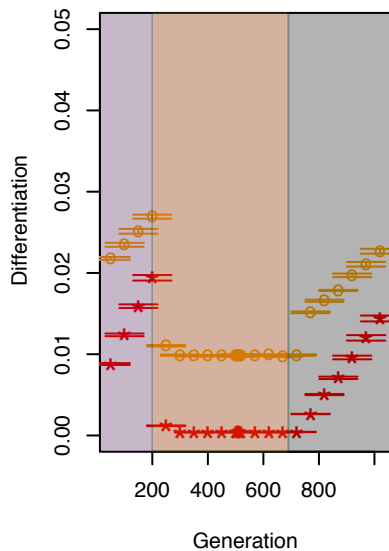**Aii**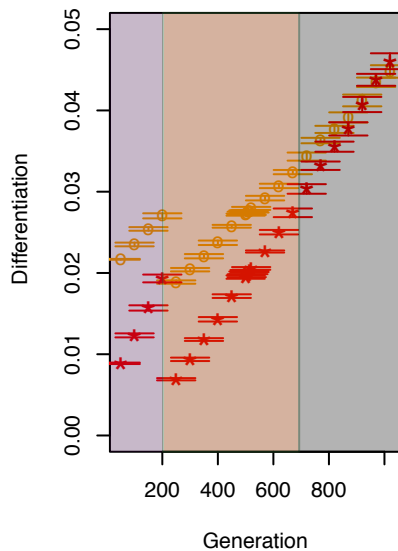

Phase I  
Phase III  
Phase IV

$F_{ST}$   
 $F_{CT}$

**Bi**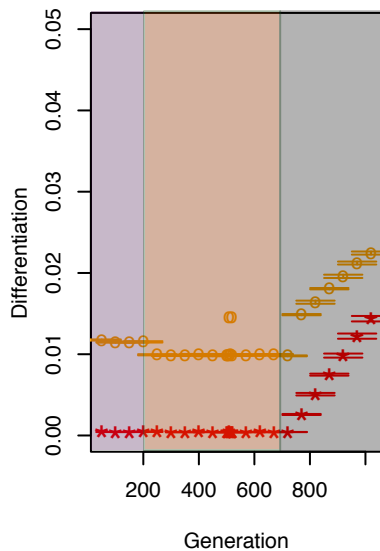**Bii**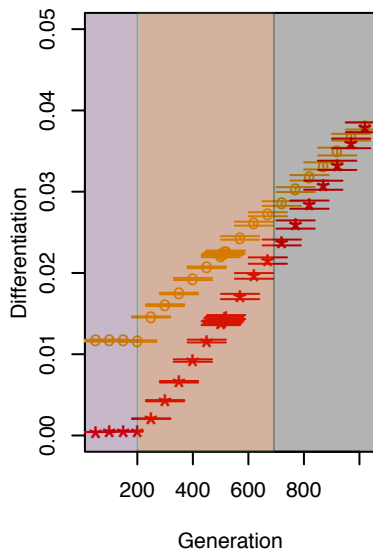**Biii**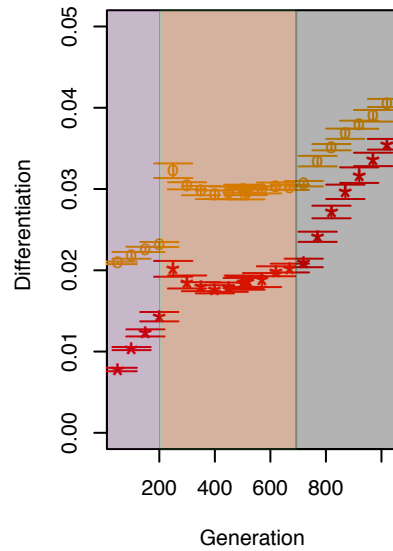

Supplement: Supplementary file 2 [file EVA-13-195-s002.pdf]

Generation 300

Ai

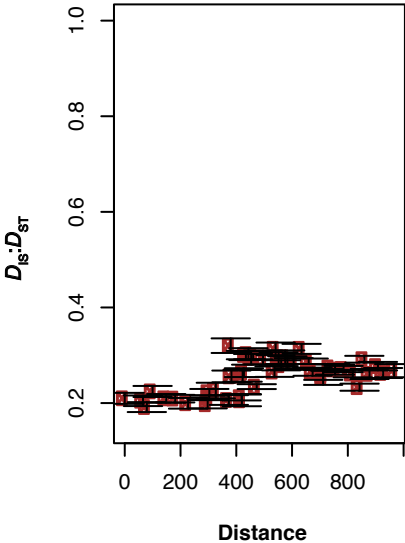

Aii

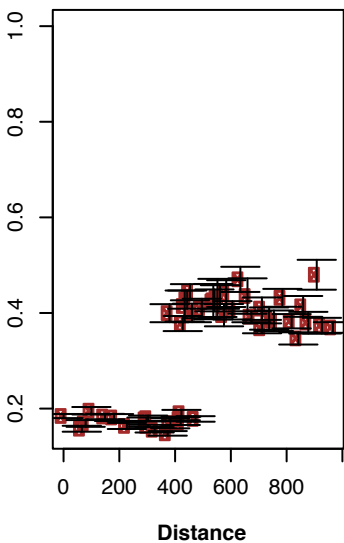

Bi

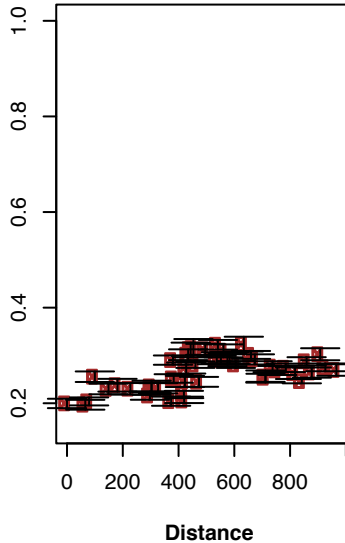

Bii

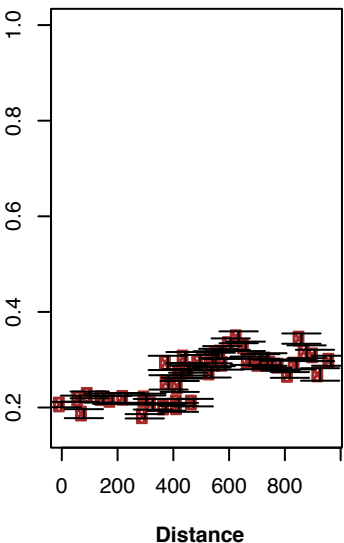

Biii

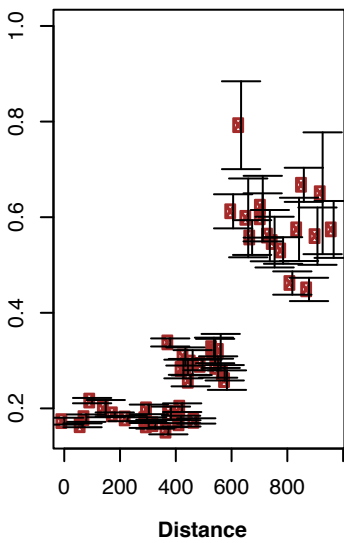

Generation 1020

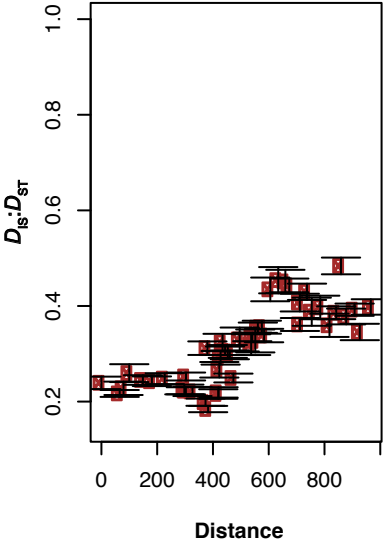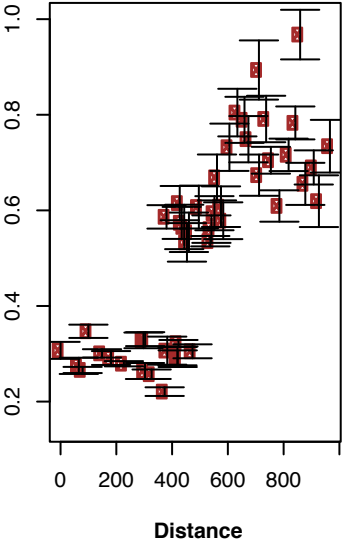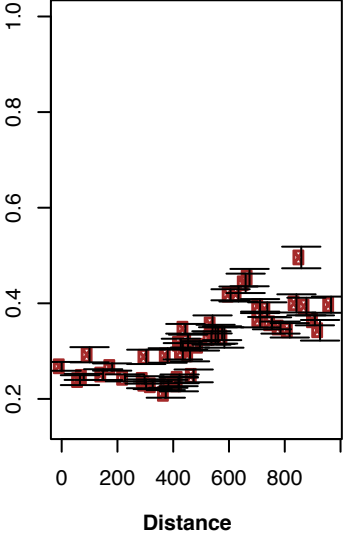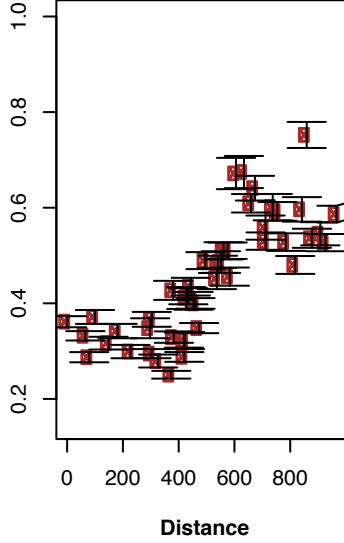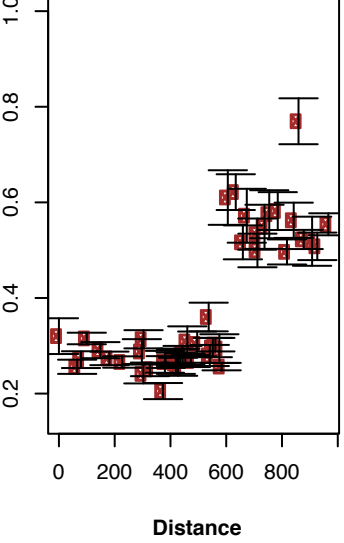

Supplement: Supplementary file 3 [file EVA-13-195-s003.pdf]
