# Supplementary material for: Tracing the footprints of a moving hybrid zone under a demographic history of speciation with gene flow
Source: Evol Appl. 2019 Apr 29;13(1):195–209. doi: 10.1111/eva.12795 (PMC6935588; doi:10.1111/eva.12795)
Supplement: Supplementary file 4 [file EVA-13-195-s004.docx]

**Table S1:** List of parameters used to set up the simulated landscape and to model gene flow among groups and between patches.

|  | **Input parameters** | **Values** |
| --- | --- | --- |
| *Patch variables* | Group sizes (number of patches) | Group1 (pure *P. strobiformis*) = 50 |
|  |  | Group2 (hybrid zone) = 40 |
|  |  | Group3 (pure *P. flexilis*) = 50 |
|  | Carrying capacity (mean ± 1sd) | Group1= 100 ± 5 |
|  |  | Group2 = 70 ± 5 |
|  |  | Group3 = 80 ± 10 |
|  | Beta-binomial parameters for allele frequency initialization | mean α Group1 = 21.78 |
|  |  | mean β Group1 = 312.04 |
|  |  | mean α Group2 = 20.28 |
|  |  | mean β Group2 = 310.56 |
|  | Mortality | Phase and scenario dependent |
| *Class variables* | Age classes | Age 0 = seeds/cones; Age 1 = trees |
|  | Individual patch initialization | At age 1 |
|  | Mortality | 100% after age 1. Non-overlapping generations |
|  | Maturation | 1 yr |
|  | Seed dispersal parameter for 2Dt distribution | 105330 and IBD |
|  | Pollen dispersal parameter for 2Dt distribution | 315990 and IBD |
|  | Reproduction | Monoecious & with replacement |
|  | Mean number of seeds | Poisson distribution (λ = 4) |
| *Run parameters* | Replicate MC runs | 12 |
|  | Generations | Total = 1020. Phase I = 200, Phase II = 20, Phase III = 500, Phase IV = 300 |
|  |  |  |

**Table S2:** Overlapping sets of 4 populations used to conduct the LD variance partitioning.

| **Set** | **Distance from south(km)** | **Pops** | **Latitude** | **Longitude** |
| --- | --- | --- | --- | --- |
| 1 | 393 | BAY | 23.56 | -104.72 |
|  |  | BAL | 26.85 | -106.81 |
|  |  | SAM | 27.33 | -107.5 |
|  |  | BAS | 27.33 | -107.5 |
| 2 | 835 | BAS | 27.33 | -107.5 |
|  |  | YEC | 28.33 | -109.02 |
|  |  | PIN | 31.93 | -109.27 |
|  |  | SCR | 32.45 | -110.78 |
| 3 | 1054 | SCR | 32.45 | -110.78 |
|  |  | LON | 32.68 | -105.64 |
|  |  | PEA | 32.92 | -108.14 |
|  |  | BRA | 32.98 | -105.71 |
| 4 | 1068 | BRA | 32.98 | -105.71 |
|  |  | COO | 33.06 | -105.63 |
|  |  | FEN | 33.23 | -105.64 |
|  |  | EAG | 33.39 | -105.73 |
| 5 | 1167 | EAG | 33.39 | -105.73 |
|  |  | WIT | 33.88 | -107.49 |
|  |  | OBS | 33.99 | -107.18 |
|  |  | CAP | 34.7 | -106.4 |
| 6 | 1370 | CAP | 34.7 | -106.4 |
|  |  | SEG | 35.16 | -108.1 |
|  |  | ROA | 35.35 | -111.62 |
|  |  | ABI | 35.37 | -111.67 |
| 7 | 1473 | ABI | 35.37 | -111.67 |
|  |  | ATH | 35.38 | -111.67 |
|  |  | KLO | 35.4 | -111.86 |
|  |  | EFS | 37.38 | -106.88 |
| 8 | 1603 | EFS | 37.38 | -106.88 |
|  |  | MSL | 37.74 | -105.45 |
|  |  | BCK | 38 | -107.66 |
|  |  | MEL | 38.07 | -110.82 |
| 9 | 1652 | MEL | 38.07 | -110.82 |
|  |  | HO | 38.07 | -105.59 |
|  |  | VC | 38.08 | -105.57 |
|  |  | BU | 39.11 | -106.16 |
| 10 | 1833 | BU | 39.11 | -106.16 |
|  |  | JEN | 39.93 | -105.66 |
|  |  | CP | 40.65 | -105.66 |
|  |  | DD | 40.81 | -104.03 |
| 11 | 1943 | DD | 40.81 | -104.03 |
|  |  | CH | 40.97 | -105.53 |
|  |  | JV | 41.03 | -106.09 |
|  |  | WY | 41.27 | -105.43 |

**Table S3**: Estimated values of *D*_IS_ and *D*_ST_ along the 1D transect for the full extent of the study design using nearly diagnostic SNPs

| **Distance (km)** | ***D*_ST_** | ***D*_IS_** | ***D*_IS_ / *D*_ST_** |
| --- | --- | --- | --- |
| 393 | 0.024 | 0.022 | 0.91 |
| 835 | 0.027 | 0.03 | 1.11 |
| 1054 | 0.024 | 0.026 | 1.08 |
| 1068 | 0.028 | 0.036 | 1.28 |
| 1167 | 0.025 | 0.025 | 1 |
| 1370 | 0.03 | 0.037 | 1.23 |
| 1473 | 0.034 | 0.058 | 1.71 |
| 1603 | 0.028 | 0.055 | 1.20 |
| 1652 | 0.03 | 0.069 | 2.30 |
| 1833 | 0.029 | 0.064 | 2.20 |
| 1943 | 0.022 | 0.041 | 1.86 |
