# Supplementary material for: Tracing the footprints of a moving hybrid zone under a demographic history of speciation with gene flow
Source: Evol Appl. 2019 Apr 29;13(1):195–209. doi: 10.1111/eva.12795 (PMC6935588; doi:10.1111/eva.12795)
Supplement: Supplementary file 5 [file EVA-13-195-s005.docx]

**Appendix I:- Details of landscape setup and genetic parameterization for the simulation in CDMetaPOP**.

***Simulation setup in CDMetaPOP:*** We simulated a landscape of size 1,024 x 1,024 units that mimicked the relative spatial extent and position of each group (*P. strobiformis* in the south, hybrid zone in the middle and *P. flexilis* in the north) in the empirical dataset (Figs. 1 & 2). Specifically, the hybrid zone occupied a smaller spatial extent (1,024 x 205) relative to the two parental species (1,024 x 410) and was located in the middle of the simulated landscape. We set the number of patches and patch size to mimic the geographical distribution and the degree of fragmentation for the three groups (Supporting information, Table S1). The location of each patch and the total carrying capacity per group were set constant throughout the simulation to avoid confounding effects of fluctuating population sizes in our assessment of hybrid zone movement. The average predicted probability of occurrence for each group, as obtained from the niche modelling results in Menon et al. (2018), was used to determine differences in regional population size. The total carrying capacity per group was then used as a proxy to reflect these differences in regional population sizes. Although niche differentiation has been demonstrated between the three groups (Menon et al., 2018), we did not incorporate environmental differences in our model since the primary focus of the analysis was to assess the influence of demographic factors on hybrid zone movement.

***Gene flow and genetic parameters:*** We used estimates of dispersal distances for seeds and pollen to parameterize gene flow parameters in our simulations. Seeds of *P. flexilis* are predominantly dispersed among populations by Clark’s Nutcrackers (Jorgensen, Hamrick & Wells, 2002; Tomback, Samano, Pruett & Schoettle, 2011) with a peak dispersal distance of 100 to 5000 m, although dispersal up to 22 km has also been observed, albeit rarely. Seeds of *P. strobiformis* are dispersed among populations by a combination of birds, small mammals and rarely by wind and gravity (Looney & Waring, 2013 and references within). Pine pollen is wind dispersed and tends to have more right-skewed dispersal kernels. Paternity analysis in *P. flexilis* has demonstrated mean pollen dispersal at 140 m (Schuster & Mitton, 2000), although other studies in *P. taeda* and *P. sylvestris* have demonstrated viable pollen dispersal up to 30 and 100 km, respectively (Williams, 2010; Robledo-Arnuncio, 2011; Kremer et al., 2012). Overall, the dispersal kernels of both seeds and pollen are Poisson distributed (Robledo-Arnuncio & Gil, 2005) and pollen dispersal tends to be more right skewed than seed dispersal (Strauss,1993). In our simulations, we divided dispersal among patches into straying and migration to mimic the different distributions of pollen and seed movement. We used the 2Dt distribution (as in Moran & Clark, 2010) to model the right-skewed nature of the dispersal kernels. We scaled the shape parameter of seed dispersal kernel to match 500 m in our simulated landscape, whereas for pollen we scaled the shape parameter by three to get a wider right-tailed distribution. In order to get most of the mating to occur within a patch and accommodate the wider right-tailed distribution for pollen, we scaled the diagonal of the dispersal matrix by 10 for seeds and 5 for pollen. Throughout our simulation workflow, dispersal (migration and straying) always occurred within groups, however, dispersal among groups was allowed to vary (details below). We initialized the simulations with 1000 biallelic loci and set their allele frequencies at *t* = 0 using a beta-binomial distribution with shape parameters determined by the equations in Balding & Nicholas (1995) using minor allele frequency estimates from Menon et al (2018) (Table S1).

**Appendix II:- Results of genetic differentiation (FST and FCT) estimates from simulations conducted in CDMetaPOP.**

The degree of genetic differentiation among patches (*F*_ST_), which includes effects for patches nested in groups as well as among groups, and only the among group differentiation (*F*_CT_) varied across the two models and across phases within each model (Table 3). During Phase I of our simulation, both measures of differentiation were higher for the model of secondary contact (Model A) relative to the model of speciation with gene flow (Model B). The level of differentiation was marginally higher during Phase III and Phase IV for Model A relative to Model B. As expected, the models with spatially restricted gene flow during Phase III (A.ii & B.ii) attained the highest level of differentiation at Phase IV. Across models, both *F*_CT_ and *F*_ST_ increased linearly from Phase III to Phase IV (Supporting information, Fig. S2). During Phase I only Model A exhibited a linear increase in *F*_ST_ and *F*_CT_ values. For all scenarios, except for A.i and B.i, the average *F*_CT_ and *F*_ST_ values at Phase III were within the range noted for those parameters in the empirical dataset (genome-wide empirical estimate: *F*_ST_ = 0.021, 95% CI: 0.008–0.031, and *F*_CT_ = 0.01, 95% CI: 0.005–0.018).
